# Human RNase3 immune modulation by catalytic-dependent and independent modes in a macrophage-cell line infection model

**DOI:** 10.1007/s00018-020-03695-5

**Published:** 2020-11-23

**Authors:** Lu Lu, RanLei Wei, Guillem Prats-Ejarque, Maria Goetz, Gang Wang, Marc Torrent, Ester Boix

**Affiliations:** 1 Department of Biochemistry and Molecular Biology, Faculty of Biosciences, Universitat Autonoma de Barcelona, Cerdanyola del Vallès, Spain; 2 Center of Precision Medicine and Precision Medicine Key Laboratory of Sichuan Province, West China Hospital, Sichuan University, Chengdu, China; 3 Present Address: College of Animal Science and Technology, Sichuan Agricultural University, Chengdu, Sichuan China

**Keywords:** Ribonucleases, Host defence, EGFR, Infection, *Mycobacterium aurum*, Respiratory syncytial virus, Transcriptome

## Abstract

The human RNase3 is a member of the RNaseA superfamily involved in host immunity. RNase3 is expressed by leukocytes and shows broad-spectrum antimicrobial activity. Together with a direct antimicrobial action, RNase3 exhibits immunomodulatory properties. Here, we have analysed the transcriptome of macrophages exposed to the wild-type protein and a catalytic-defective mutant (RNase3-H15A). The analysis of differently expressed genes (DEGs) in treated THP1-derived macrophages highlighted a common pro-inflammatory “core-response” independent of the protein ribonucleolytic activity. Network analysis identified the epidermal growth factor receptor (EGFR) as the main central regulatory protein. Expression of selected DEGs and MAPK phosphorylation were inhibited by an anti-EGFR antibody. Structural analysis suggested that RNase3 activates the EGFR pathway by direct interaction with the receptor. Besides, we identified a subset of DEGs related to the protein ribonucleolytic activity, characteristic of virus infection response. Transcriptome analysis revealed an early pro-inflammatory response, not associated to the protein catalytic activity, followed by a late activation in a ribonucleolytic-dependent manner. Next, we demonstrated that overexpression of macrophage endogenous RNase3 protects the cells against infection by *Mycobacterium aurum* and the human respiratory syncytial virus. Comparison of cell infection profiles in the presence of Erlotinib, an EGFR inhibitor, revealed that the receptor activation is required for the antibacterial but not for the antiviral protein action. Moreover, the DEGs related and unrelated to the protein catalytic activity are associated to the immune response to bacterial and viral infection, respectively. We conclude that RNase3 modulates the macrophage defence against infection in both catalytic-dependent and independent manners.

**Electronic supplementary material:**

The online version of this article (10.1007/s00018-020-03695-5) contains supplementary material, which is available to authorized users.

## Introduction

Human RNase3, also known as the Eosinophil Cationic Protein (ECP), is a member of the ribonuclease A superfamily. It is a small and highly cationic protein that participates in the immune defence response. RNase3 is mainly expressed in eosinophils, the mature protein is stored in the eosinophil secondary granules and is secreted upon infection and inflammation [1–3]. Together with its main production source, the protein is also reported to be expressed in other leukocytes, such as neutrophils and macrophages [4]. The importance of the selective expression and secretion of RNase3 has been emphasized due to its association with multiple diseases, such as asthma, intestinal tract inflammation or autoimmune disorders [1, 2, 4, 5]. The protein is routinely used as a clinical diagnostic marker of eosinophil activation during inflammatory processes [2]. Secretion of RNase3 is also induced by damaged epithelia and the protein can participate in tissue healing and remodelling [4]. In addition, RNase3 expression is induced by infection and might contribute to the protection of biological fluids. The protein exhibits antimicrobial activity against a wide range of microorganisms, such as bacteria, yeast, viruses, and parasites [3, 6–10]. Abundance of surface-exposed cationic and hydrophobic residues can mediate the protein binding and subsequent destabilization of bacterial membranes through a carpet-like mechanism characteristic of many host defence antimicrobial peptides (AMPs) [10–12]. Although the protein triggers the pathogen death mainly through a direct mechanical action at the cell envelope, the targeting of intracellular components, such as nucleic acids cannot be disregarded. Indeed, RNase3 internalization is observed in treated yeast cells and protozoa [8, 13]. Besides, RNase3 can enter the macrophage and eradicate the intracellular dwelling bacteria [14]. On the other hand, the protein antiviral activity on single stranded RNA viruses has been directly correlated to its ribonucleolytic action [9]. Together with the RNase3 direct antimicrobial activity, a series of evidences illustrated that RNase3 also plays an immunomodulatory role during host defence [2, 4]. Early studies showed that RNase3 can activate rat mast cell and induce histamine release, mediating the cross talk between mast cells and eosinophils [15]. RNase3 displays remodelling activity partly mediated by inducing the expression of epithelial insulin-like growth factor 1 (IGF1) [5] and the fibroblast chemotaxis can be enhanced by RNase3 release into the injured tissue site [2, 16]. Recently, we observed that RNase3 expression in macrophages is regulated by mycobacteria infection. Moreover, we demonstrated the protein ability to enter macrophage cells and induce autophagy, contributing thereby to the eradication of the intracellular infection [14]. However, how RNase3 regulates the macrophage function and whether the protein immunomodulatory properties are dependent on its ribonucleolytic activity remains to be clarified.

In the present study, we have investigated how RNase3 modulates the transcriptome profile of the macrophage cell. Towards this end, we used the human monocytic cell line THP1 derived to macrophage and applied the next-generation RNAseq methodology to analyse the cell response to protein treatment. In addition, we explored the potential contribution of the protein catalytic activity on macrophages using a mutant enzymatically defective mutant (RNase3-H15A). Notably, this is the first systematic study to investigate the biological function of human RNase3 in macrophages by the whole transcriptome analysis. Complementarily, we built an RNase3 overexpression THP1 cell line by CRISPRa to validate the transcriptome results and evaluated the direct contribution of the endogenous protein in the eradication of intracellular infection by *M. aurum* and the human respiratory syncytial virus (RSV)*.* The results revealed that RNase3 can inhibit both bacterial and viral infection through the modulation of the macrophage immune response in both ribonucleolytic-dependent and independent ways.

## Materials and methods

### Cell culture

Human THP1 cells (NCTC #88081201) were maintained or passaged in 25 or 75 cm^2^ tissue culture flasks (BD Biosciences) using RPMI-1640 (Lonza, BE12-702F) medium with 10% heat-inactivated foetal bovine serum (FBS) at 37 °C, humidified 5% CO_2_ conditions. THP1 cells used in this study were controlled below passage 25. THP1 cells were treated with 50 nM phorbol myristate acetate (PMA, Sigma-Aldrich, P8139) for 48 h to induce differentiation into macrophage-like cells and allowed to rest for 24 h before further treatment.

### Recombinant protein expression and purification

RNase3 and RNase3-H15A recombinant proteins were produced as previously reported [8]. Briefly, *E. coli* BL21(DE3)/pET11c cells were induced by 1 mM Isopropyl β-d-1-thiogalactopyranoside (IPTG, St. Louis, MO, USA) and the inclusion bodies enriched pellet was resuspended in 80 mL of 10 mM Tris–HCl pH 8.5, 2 mM EDTA and left incubating 30 min with 40 µg/mL of lysozyme prior to sonication. Following, the sample was centrifuged at 30.000 × g for 30 min at 4 °C and the pellet was resuspended in 25 mL of the same buffer with 1% triton X-100 and 1 M urea and was left stirring at room temperature for 30 min and then centrifuged 30 min at 22.000 × *g*. Following, 200 mL of 10 mM Tris–HCl pH 8.5, 2 mM EDTA was added to the pellet, and then the sample was centrifuged at 22.000 × *g* for 30 min (4 °C). The resulting pellet solubilized in 6 M guanidine hydrochloride and rapidly 80-fold diluted in the refolding buffer was left in gentle stirring for 48–72 h at 4 °C. The folded protein was then concentrated, dialyzed against the chromatography buffer and purified first by cation chromatography using a Resource S (GE Healthcare Life Sciences) column and then by reverse phase chromatography on a Vydac C4 (ThermoFisher Scientific) column. Sample purity (> 99%) was checked by SDS-PAGE and MS spectrometry.

Complementary, recombinant proteins corresponding to RNase3 wild-type (rRNase3-97^Arg^, R97) and the arginine to threonine variant (rRNase3-97^Thr^, T97), expressed in insect cells using the pFASTBAC baculovirus expression system, were purchased from *Diagnostics Development* company*.* Both protein variants had been purified and quantified as previously described [17].

### Treatment of THP1 cells derived to macrophages with RNase3 and RNase3-H15A

THP1 cells were differentiated into macrophage by 50 nM of PMA treatment as previously described [14]. The cells were then washed three times with prewarmed PBS and replaced with fresh RPMI + 10%FBS medium. THP1-derived macrophages were then treated with 10 μM of the recombinant proteins, wild-type (wt) RNase3 or the RNase3-H15A mutant. Previous work confirmed that the selected protein concentration ensured an effective antimicrobial activity and was non-toxic to the macrophage cells [14]. After 4 and 12 h of treatment, the cells were washed three times with pre-warmed PBS and collected for further RNA extraction. Three biological repeats were carried out for each experiment. Alternatively, prior to treatment with the protein, cells were pre-incubated with a monoclonal antibody against the extracellular domain of the EGFR, anti-EGFR Ab Cetuximab*,* Abcam) at 10 μg/mL for 1 h at 4 °C, as previously described [18].

### RNA isolation and sequencing

Total RNA was extracted using the mirVanaTM miRNA Isolation Kit (Ambion, Life Technologies, AM1560) as described by the manufacturer. RNA purity was determined by spectrophotometry and RNA integrity (RIN) was analysed using Agilent 2100 Bioanalyzer (Table S1). Following RNA extraction, total RNAs were submitted to the CNAG-CRG Sequencing Service (*Centre for Genomic Regulation*, Barcelona) for cDNA library preparation,polyA enrichment and NGS sequencing. Sequencing libraries were prepared according to protocols provided by Illumina. 50 bp-long single-end sequencing was carried out in an Illumina HiSeq2500 sequencer with a depth of > 20 million reads per sample. Raw sequence reads have been deposited in the NCBI Sequence Read Archive (SRA) under accession number PRJNA574982.

### Transcriptome analysis

FastQC was used to carry out the quality assessment of reads, assessing the distribution of phred quality scores and mean percentage GC content across each read. Reads were aligned to the latest human genome assembly from the Genome Reference Consortium (GRCh38) using HISAT2 [19]. Aligned reads were stored in the SAM file format. StringTie was used to assemble the alignments into transcripts and estimate the expression levels of all genes and transcripts [19]. Low expression (sum count less than 10) and non-coding genes were filtered out using biomaRT [20] before passing to the Bioconductor package DESeq2 [21]. The resulting *P* values were adjusted using Benjamini and Hochberg’s approach for controlling the false discovery rate (FDR). Genes with an adjusted *P* value (*Q* value) < 0.01 & log2FC absolute value > 1 found by DESeq2 were assigned as differentially expressed genes (DEGs).

GO enrichment and KEGG pathway enrichment analysis of the differential expression of genes across the samples was carried out using the clusterProfiler R package [22]. Protein–protein interactions between DEGs were analysed using NetworkAnalyst which integrates the experimentally validated interactions database, InnateDB database [23]. The network was visualized using Cytoscape software (https://cytoscape.org).

### Molecular modelling

HADDOCK2.2 (Utrecht Bioinformatics Center, University of Utrecht) was applied to perform the modelling of the protein complexes and predict the associated free energies [24, 25].

### Construction of an RNase3 overexpression THP1 cell line

We applied CRISPRa to activate the endogenous expression of RNase3 in THP1 [26]. Five distinct sgRNAs targeting the region from 100 to 500 bp relative to the transcription start sites (TSS) of the RNase3 gene were designed using Cas9 Activator Tool (https://sam.genome-engineering.org/database/) [26]; the sgRNA sequence is listed in Table S2. The pLenti239G plasmid, used to co-express the dCas9-VP64 fusion protein and EGFP, was constructed by inserting the T2A-EGFP cassette from the LentiCRISPRv2-GFP-puro (gifted by Manuel Kaulich) in place of the T2A-BSD cassette of the lenti-dCAS-VP64-Blast (Addgene61425, gifted by Manuel Kaulich). The pLenti239R, used to co-express the gRNA, cherry red fluorescence protein, and puromycin resistance gene, was constructed by displacing the Cas9-T2A-EGFP of LentiCRISPRv2-GFP-puro (gifted by Manuel Kaulich) with Cherry red fluorescent gene (gifted by Marcos Gil García, UAB). The sgRNA sequences were inserted in pLenti239R at downstream of the U6 promoter using the BbsI cloning sites. The correct construction of the plasmids was validated by Sanger sequencing.

Following, HEK293T cells were used for lentiviral production. HEK293T cells were maintained in DMEM + 10% FBS complete medium in a 5% CO_2_ humidified incubator at 37 °C. Cells were co-transfected with psPAX2 (Addgene#35002, gifted by Marina Rodriguez Muñoz) packaging plasmid, pMD2.G (Addgene#12259, gifted by Marina Rodriguez Muñoz) envelope plasmid, and pLenti-239G (encoding dCAS9 and eGFP) or pLenti-239R (encoding sgRNA and Cherry red fluorescent marker) using calcium phosphate precipitation protocol [27]. The transfection medium was replaced with fresh medium following overnight incubation. Supernatants were collected 24, 48 and 72 h after transfection, centrifuged to remove cell debris, filtered using 0.45 μm filters and concentrated using the PEG6000 precipitation method [28]. Viral pellets were resuspended in PBS, aliquoted and stored at – 80 °C until use. THP1 monocytes were infected with 20 μL concentrated lentivirus in the presence of 8 μg/mL polybrene and incubated overnight. Next day, the cells were replaced with fresh medium and cultured for 72 h. Fluorescence positive monocytes were checked by fluorescence microscopy and then sorted by Cell sorter. The fluorescence positive cells were evaluated by suspending the cells in PBS and fixing them with 2% paraformaldehyde for 10 min prior to flow cytometer. The fluorescence positive cells were sorted into single cells by Cell sorter BD FACSJazz.

### Real-time qPCR assays

Following the treatment of THP1-derived macrophages with recombinant RNase3 proteins the transcriptional expression profiles of selected genes were measured by RT-qPCR with *GAPDH* as the internal control gene. Recombinant EGF (ThermoFisher) was used as a positive control in some assays. Primers used for RT-qPCR validation are listed in Table S2. The same total RNA was reverse transcribed into cDNA using iScriptTM cDNA Synthesis Kit (Bio-Rad, 1708891). RT-qPCR assays were performed in 20 μL using the iTaq Universal SYBR Green Supermix (Bio-Rad, 1725121) according to the manufacturer's instructions. The reactions were conducted using CFX96 Real-Time PCR detection system (Bio-Rad, Hercules, CA, USA) under the following conditions: 95 °C for 2 min; 40 cycles of 95 °C for 15 s and 60 °C for 30 s; melting curve generation (60–95 °C). The relative expression ratios were calculated using the 2 − ΔΔCT method [29, 30].

### Western blot

For the western blot assays to detect RNase3, cells were harvested and lysed with RIPA buffer. Samples to analyse pMAPK were extracted with TRIzol™ (ThermoFisher) and resuspended in 1 M of urea, 2% of SDS and 5 mM EDTA. The samples were separated by 10% SDS-PAGE. Then, the samples were transferred to polyvinylidene difluoride membranes, blocked with either 5% non-fat milk or BSA in TBST, and incubated with anti-RNase3 primary antibody (Abcam, ab207429), or anti-p44/42 MAPK (Cell Signaling, 9106S) overnight at 4 °C. After washing, the membranes were treated for 1 h at room temperature (RT) with horseradish peroxidase (HRP)-conjugated goat anti-rabbit IgG (Sigma Aldrich, 12-348) or HRP-conjugated horse anti-mouse IgG (Cell Signaling, 7076P2), respectively. Finally, the membranes were exposed to an enhanced chemiluminescent detection system (Supersignal West Pico Chemiluminescent Substrate, ThermoFisher Scientific, 32209) for detection. As a control, GAPDH was detected with chicken anti-GAPDH antibodies (Millipore).

### Macrophage infection by mycobacteria and CFU assay

Infection of THP1 cells derived to macrophage by mycobacteria was performed as previously described [14]. *M. aurum* was purchased from the UK National Collection of Type Cultures (NCTC). Cells cultures of *M. aurum* (NCTC, 10437 were grown in Middlebrook (MB) 7H9 broth (BD Biosciences, 271310) enriched with 10% (v/v) albumin/dextrose/catalase (ADC; BD Biosciences, 212352) containing 0.05% Tween 80, and in MB7H10 (BD Biosciences, 262710) with 10% (v/v) oleic acid/albumin/dextrose/catalase (OADC; BD Biosciences, 212240) for semi-solid agar growth at 37 °C. Stock cultures of log-phase cells were maintained in glycerol (25% final concentration) at − 80 °C. The bacteria were vortexed and sonicated using ultrasound sonication bath to obtain a single cell suspension, and then the bacterial concentration was determined by measuring the optical density (OD) of the culture at 600 nm (OD = 10^9^ CFU/mL). Mid-log phase *M. aurum* cells, harvested in RPMI-1640 complete medium, were co-cultured with macrophages at a multiplicity of infection (MOI) of 10:1 and were incubated at 37 °C for 3 h, then were washed three times with PBS and replaced with fresh media supplied with 50 μg/mL gentamycin (Apollo Scientific, BIG0124) to remove extracellular mycobacteria during further treatment.

Colony forming units (CFU) counting assay was applied to compare the infectivity and living rate of *M. aurum* toward THP1-derived macrophages with or without RNase3 overexpression. 2 × 10^5^ THP1 cells were seeded in 24-well plates per well and induced to macrophages by 50 nM of PMA treatment. Next, log-phase cultures of *M. aurum* (OD_600_ ~ 1) were diluted to 2 × 10^6^ CFUs/mL and were used to infect macrophages derived from wild-type macrophage (WT) or RNase3-overexpression macrophage cells (OX) at a multiplicity of infection of 10:1 at 37 °C for 3 h. Then the cells were washed 3 times with PBS and replaced with fresh media supplied with 50 μg/mL gentamycin to remove extracellular mycobacteria, referenced as the “0 h post-infection” time point. At 0 h, 24 h, 48 h, and 72 h, the cells were washed, collected, lysed with distilled water, and plated in 10 mm petri dish containing MB7H10/OADC/agar. The CFU were counted after 2 weeks. Five independent experiments were conducted for this assay.

### Macrophage infection by RSV virus and viral quantification

Human respiratory syncytial virus (RSV, ATCC, VR-1540) stock was ordered from ATCC. Hela cells were used to produce RSV under biosafety level II conditions [31]. Briefly, Hela cells are plated in 75 cm^2^ culture flask and incubated at 37 °C in DMEM + 10% FBS until they are approximately 50% confluent. The cells were then washed and infected with RSV stock under a MOI of 0.1. After 3 h infection, the cells were washed and replaced with fresh medium (DMEM + 10%FBS) and incubated for 4 days at 37 °C , 5% CO_2_. The cells and the virus suspension were collected when the cytopathology appeared, with scraping and vortexing of the cells to release more viral particles. The virus suspension was centrifuged for 10 min at 1800 g to remove the cell debris. The virus suspension without cell debris were concentrated using Amicon Ultra-15 centrifugal filters with 100 kDa cut-off (Millipore, UFC910024). The produced viruses were titrated by measuring the median tissue culture infectious dose (TCID50) in HEK293T cells [32].

Before RSV infection, THP1 cells were induced to macrophage by 50 nM of PMA treatment for 48 h. Cells were washed three time with prewarmed PBS and replaced with fresh RPMI + 10% FBS medium for 24 h incubation. After that, macrophages were washed and incubated with RSV, mixing every 15 min for the first 2 h. All virus treatment tests were performed using RSV at a MOI of 1 TCID50/cell.

RSV was detected by RT-qPCR [33]. After the indicated post-infection time, the extracellular RSV virus was collected by the PEG6000 precipitation methodology [28, 34] and intracellular RSV virus were collected by lysing the macrophage cells with the lysis buffer from mirVanaTM miRNA Isolation Kit (Ambion, Life Technologies, AM1560). Total RNA from RSV-infected macrophage cells as well as stock virus was extracted using mirVanaTM miRNA Isolation Kit according to the manufacturer’s instructions. cDNA was synthesized using iScriptTM cDNA Synthesis Kit (Bio-Rad, 170-8891). The synthesis was performed using random hexamers, starting with 1 μg of total cell RNA. The RT-qPCR was performed using ddPCR ™ Supermix for Probes (Bio-Rad, 1863024). Samples with a cycle threshold value of more than 40 were recorded as negative. A standard curve was prepared using serially diluted RNA extracts from a known quantity and used to quantify RSV as TCID50/mL. In parallel with the RSV probe assays, an endogenous glyceraldehyde-3-phosphate dehydrogenase (*GAPDH*) control was used for relative quantification of the intracellular virus. The primers and probe used are listed in Table S2.

### Cell viability assay

Cell viability was measured using MTT assay as previously reported [35]. After each time point of post of infection, cells were incubated with 100 μL of MTT reagent. The media were then removed and 200 μL of DMSO was added per well. The absorbance of the formazan was determined at 570 nm in an ELISA reader.

## Results

### Comparative transcriptome analysis of THP1-derived macrophages treated with wild-type RNase3 and catalytic-defective RNase3-H15A mutant

To characterize the immunomodulatory properties of RNase3 on macrophages we incubated THP1-derived cells in the presence of the protein and analysed the cell transcriptome at 4 h and 12 h incubation time. Complementarily, to explore the contribution of the protein enzymatic activity to its immune-regulatory action, we compared the macrophage-induced response by wild-type (wt) RNase3 with a protein mutant variant (RNase3-H15A) devoid of ribonucleolytic activity. Our previous work indicated that the H15A substitution fully abolishes the RNase3 catalytic activity without any alteration of the 3D protein structure [8]. RNase activity contributes to the protein antifungal and antiviral properties [8, 36]. However, some antimicrobial properties of RNase3 were reported to be independent on its catalytic action [12, 37, 38]. Besides, we recently observed that both wild-type RNase3 and H15A mutant can induce the autophagy pathway and mediate the eradication of intracellular mycobacteria within macrophages [14]. Therefore, we decided to carry out a whole transcriptome analysis to identify the distinct cell pathways associated to the protein action.

For each condition, three biological replicates were prepared for control, wt RNase3 and RNase3-H15A treatment of THP1 macrophage-derived cells. A total of 511 million reads (mReads) were obtained, providing about 28 mReads per sample. Approximately, 96% of the reads were uniquely mapped to the *Homo sapiens* genome (Table S3). Expression of 14801 protein coding genes were detected in at least one individual (filtered by sum count more than 10) (Additional file 1). The Principal Components Analysis (PCA) plot shows tight clustering within the control group and the protein treated groups (either RNase3 or RNase3-H15A treatment) for mRNA expression (Figure S1). Additionally, RNase3- and RNase3-H15A-treated samples clustered separately from each other, indicating temporal alterations in gene expression driven by different protein treatment.

**Fig. 1:**
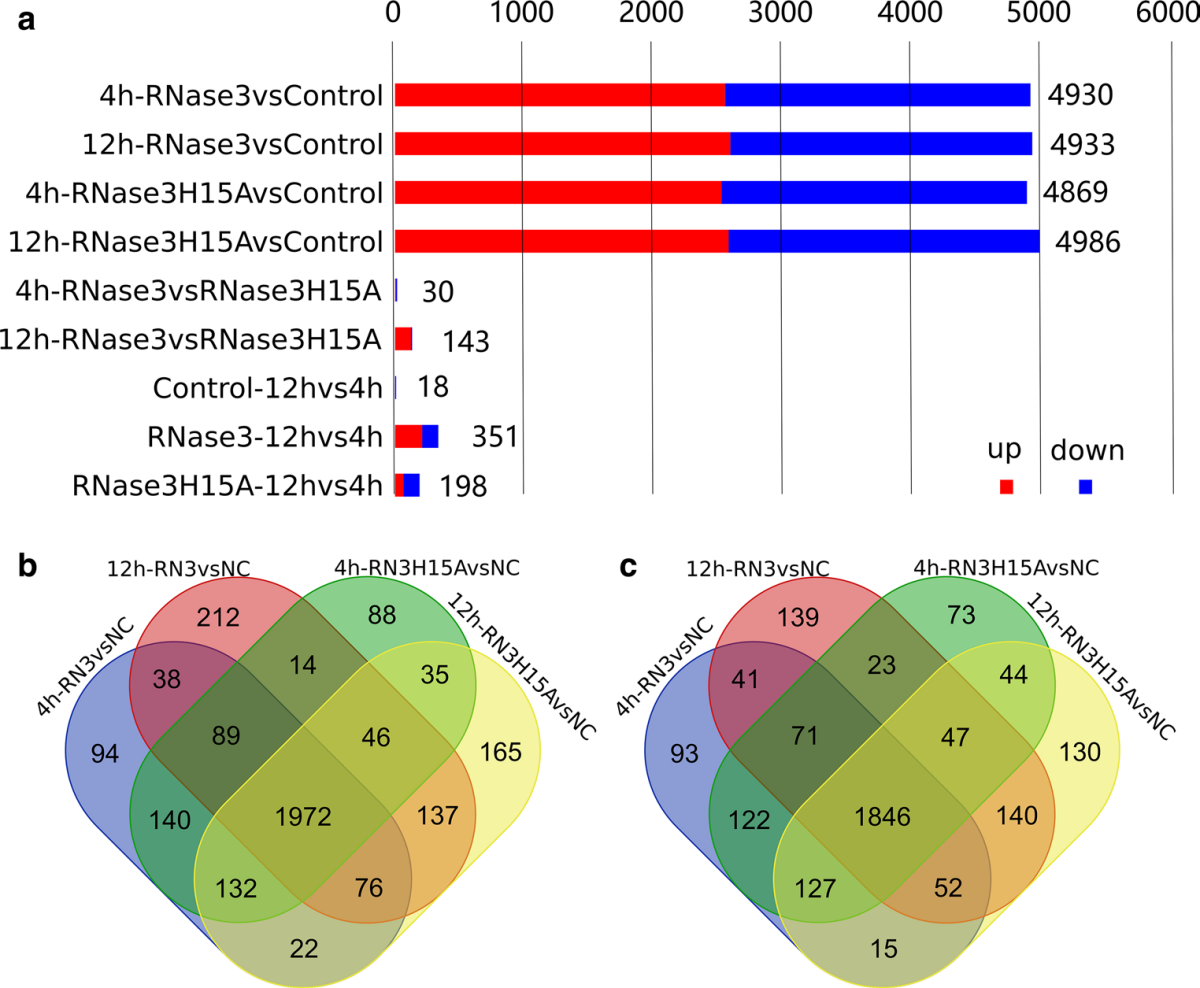
DEseq2 analysis Count of differential expressing genes (DEGs). **a** Genes with an adjusted *P* value < 0.01 & log2FC absolute value > 1 found by DESeq were assigned as differentially expressed (DE) genes. The common DEGs responded to RNase3 and RNase3-H15A treatment of THP1-derived macrophages at 4 h and 12 h was identified by overlapping. Venn plot of the DEGs from paired comparison: **b** common up-regulated and **c** common down-regulated DEGs

Following, we used DESeq2 to identify Differently Expressed Genes (DEGs) for the following conditions: Genes with an adjusted P value < 0.01 & log2FC absolute value, (i) RNase3 versus control, (ii) RNase3-H15A versus control, and (iii) RNase3-H15A versus RNase3. We identified 4930 (2563 up-regulated and 2367 down-regulated) and 4933 (2584 up-regulated and 2349 down-regulated) DEGs at 4 h and 12 h, respectively, between RNase3-treated cells and untreated control cells. When comparing RNase3-H15A-treated cells to untreated control cells at 4 h and 12 h, respectively, 4869 (2516 up-regulated and 2353 down-regulated), and 4986 DEGs were identified (2585 up-regulated gene and 2401 down-regulated) (Fig. 1a). Next, we identified DEGs by comparing 4 h and 12 h timing for each treatment, 18 (10 up regulated and 8 down regulated), 351 (223 up-regulated and 128 down-regulated) and 198 (81 up-regulated and 117 down-regulated) DEGs were found in control, RNase3, and RNase3-H15A treatment as a function of time, respectively. The small amount of DEGs between 4 and 12 h found in control samples indicated that the biology status of the cells has been stable during all the experiment. A direct comparison between RNase3 and RNase3-H15A-treated cells found 30 (8 up-regulated and 22 down-regulated) and 143 (10 up-regulated and 133 down-regulated) DEGs at 4 h and 12 h post of treatment, respectively. All the identified DEGs are listed in Additional file 2.

### RNase3 modulates the macrophage global innate immune response in a ribonuclease independent manner

Given the high overlapping group of DEGs between RNase3 and RNase3-H15A-treated cells either at 4 h or 12 h (Fig. 1b, c), we sought to identify the genes with similar response profiles. Profiles with overlap between RNase3 and RNase3-H15A-treated cells, comprising a total of 3818 genes, were identified to conform the core response of macrophage modulated by both RNase3 and RNase3-H15A. Core response genes not related to the protein ribonucleolytic activity are listed in Additional file 3 (1972 and 1846 are up-regulated and down-regulated, respectively; see Fig. 1b, c).

**Fig. 2:**
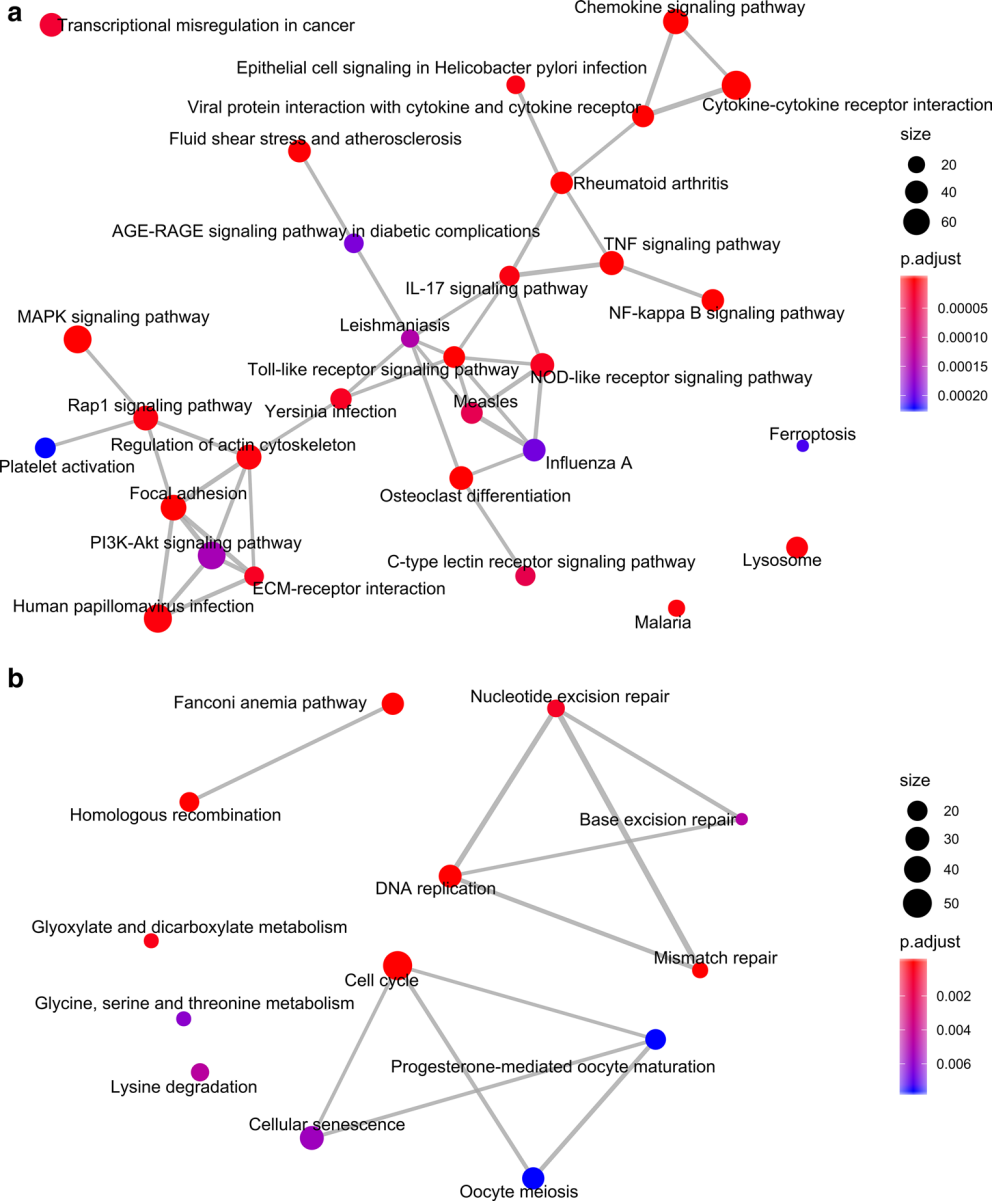
KEGG pathway enrich map of the common response DEGs to RNase3 and RNase3-H15A treatment of THP1-derived macrophages. KEGG pathways enriched by **a** common up-regulated DEGs and **b** common down-regulated DEGs

**Fig. 3:**
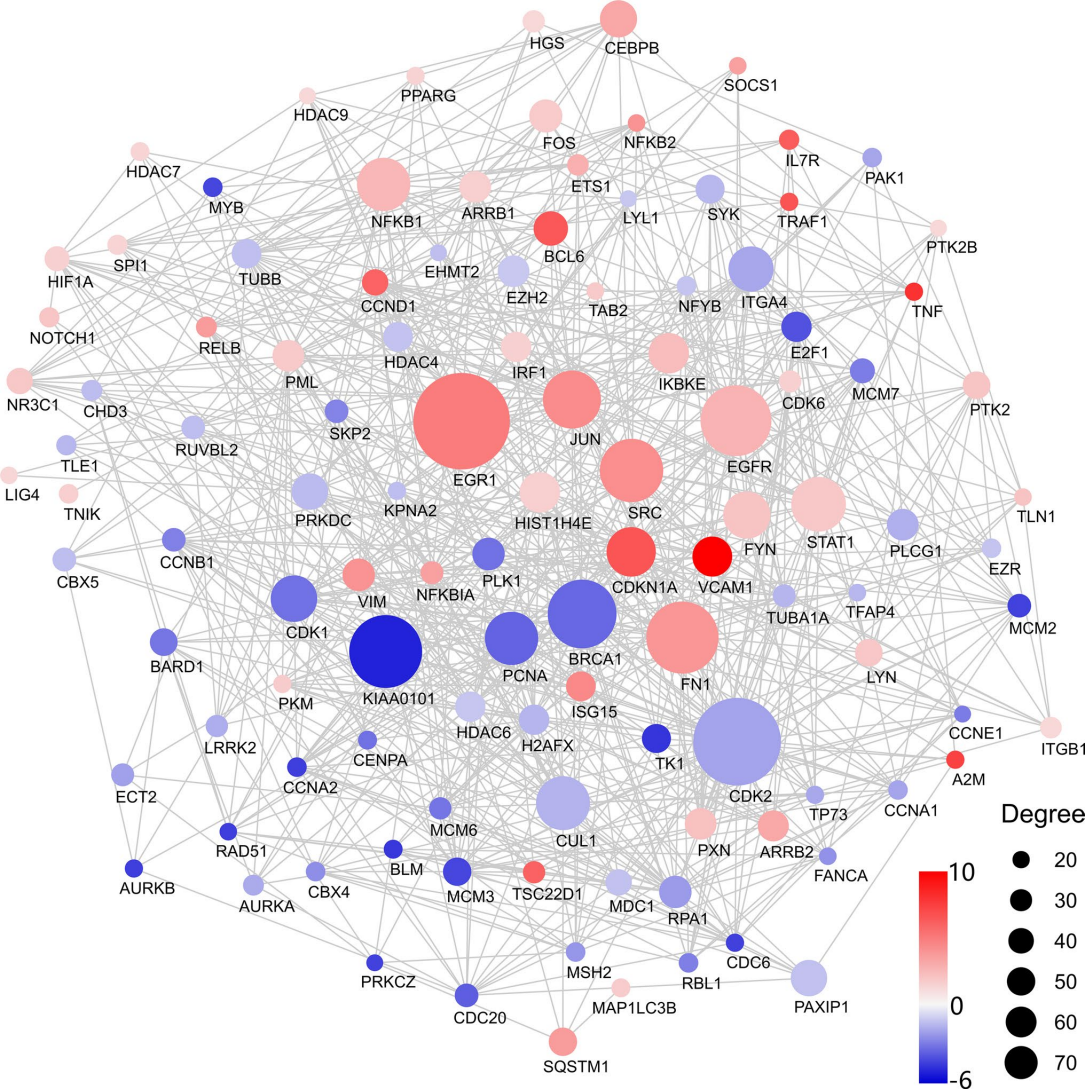
Protein–protein interaction (PPI) analysis of common response DEGs. The common DEGs were studied using the NetworkAnalyst3.0 online tool. The interaction between each protein pair is indicated with lines, the circle size is proportional to interaction degree. Only the genes with more than 25 interactions were shown. The colour bar indicated the log2FC of each gene

KEGG pathway enrichment of the up-regulated DEGs indicated that both RNase3 and RNase3-H15A treatment triggered the cells overall immune response. In total, 58 KEGG pathways were significantly enriched (padj < 0.01) (see Fig. 2a and Additional file 4). The top ten listed pathways included TNF signal pathway, Cytokine–cytokine receptor interaction, NF-kappa B signal pathway, Chemokine signalling pathway, Toll-like receptor signal pathway and MAPK signalling pathway. To examine more closely the correlations between the genes of the core response to RNase3 and RNase3-H15A treatment, we incorporated a network-based approach using the NetworkAnalyst3.0 with InnateDB database [23]. Ranked by connectivity and betweenness centrality, *EGR1*, *FN1*, *EGFR*, *SRC*, *JUN*, *STAT1*, *NFkB1* and *CDKN1A* were identified as the primary hub and bottleneck genes of macrophages induced upon wt RNase3 and RNase3-H15A exposure (Fig. 3, Additional file 5). Among them, the Epithelial Growth Factor Receptor (EGFR) is one of the main up-regulated connector, together with EGR1, SRC, JUN, STAT1 and NFkB1. Moreover, EGFR shares direct or indirect interactions with all the other central hub genes: EGR1, SRC, STAT1, NFkB1, and JUN and SRC transcription regulator. EGFR activation positively regulates transcription factors that mediate the inflammatory response: STAT1, responding to cell stress injuries [39]; NF-kB, which controls pro-inflammatory cytokine production and cell survival [40], SRC, a regulatory protein kinase [41] and EGR1, activated during tissue remodelling. Therefore, we can conclude from the network analysis results that EGFR is the main hub of the core response induced by both wt RNase3 and the RNase3-H15A mutant.

Next, we explored the down-regulated DEGs in THP1-derived macrophages upon exposure of both wt and the catalytic-defective RNase3. We identified 1846 DEGs counts associated to RNase3/RNase3-H15A common response (Fig. 1c), 13 pathways were significantly enriched (Fig. 2b; Additional file 4). Down-regulated pathways include cell cycle, DNA replication, homologous recombination and mismatch repair among others, which are indicators of a cellular growth inhibition and duplication arrest response. To note, *CDK2*, *KIAA0101*, *BRCA1*, *CUL1*, *PCNA* and *CDK1* were considered the hub and bottleneck genes by network analysis (Fig. 3, Additional file 5). As illustrated by their key connectivity we can highlight *CDK2* and *KIAA0101*, followed by *BRCA1*, *CUL1* and *PCNA*, and last *CDK1*. Interestingly, BRCA1, CDK1 and CDK2 are also associated to the EGFR pathway [42]. Moreover, PCNA is involved in the direct response to EGFR related to DNA damage [43].

**Fig. 4:**
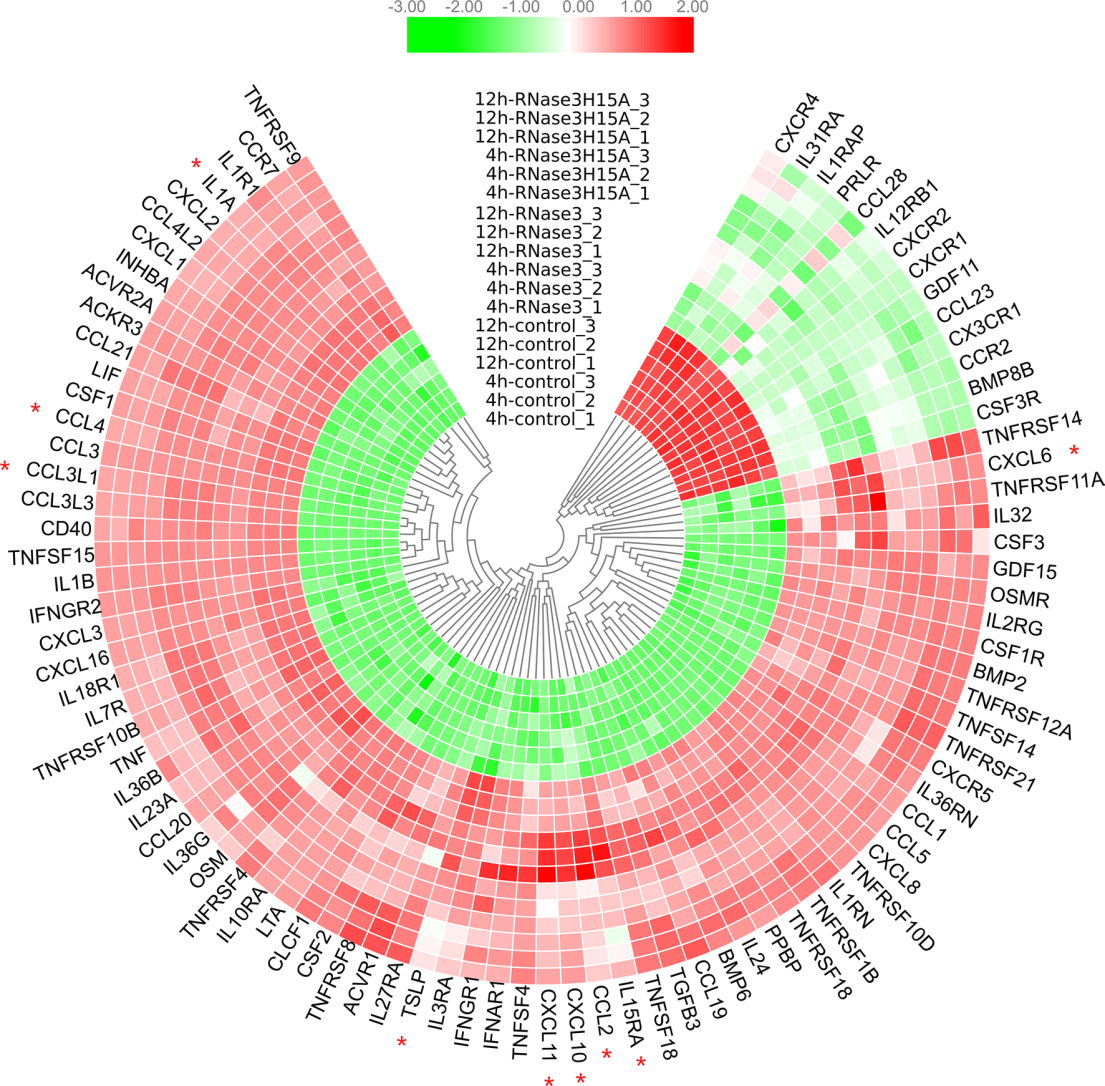
Heat-map of DEGs enriched in cytokine–cytokine receptor interaction pathways. Pairwise comparison for control, RNase3 and RNase3-H15A at 4 h and 12 h expression profiles was performed. Colour bar indicates the Log2FC for each gene. Red stars indicate correlation with the protein catalytic activity

Following, we studied the macrophage cell response upon protein exposure as a function of time. Considering that cytokine–cytokine receptor interaction is within the top common enriched pathway upon wt RNase3 and RNase3-H15A treatment, with more than 78 genes significantly up-regulated (Fig. 2a, Additional file 4), we decided to compare their dynamic changes at 4 h and 12 h time points. Compared to control, RNase3 or RNase3-H15A triggered a fast inflammatory response of the macrophage cell, as witnessed by a significant up-regulation of 20 hallmark inflammatory genes, such as *TNFα*, *IL1β*, *CD40*, *CXCL8* and *CCL4L2*. Moreover, 19 hallmark TNFα-signalling via NFkB genes were also significantly upregulated, such as *CXCL1*, *CXCL2*, *CXCL3*, *IL7R*, *LIF*, *CCL3L1*, *CCL4* and *TNFRSF12A* (see Additional file 6). Likewise, we identified a group of significantly down-regulated genes, such as *CXCR4*, *IL31RA*, *IL1RAP*, *PRLR*, *CCL28*, *IL12RB1*, *CXCR2*, *CXCR1*, *GDF11*, *CCL23*, *CX3CR1*, *CCR2*, *BMP8B*, and *CSF3R*. According to the timeline, the gene counts of most pro-inflammatory genes decreased by 12 h in comparison to a 4 h (Fig. 4), indicating that the pro-inflammatory effect triggered by RNase3 is mainly a short-term effect and is reduced upon time. Notwithstanding, a small group of genes associated to inflammation were upregulated after a 12 h exposure time, such as CCL2, CXCL10 and CXCL11. Notably, together with pro-inflammatory cytokines, we can identify up-regulated chemokines related to leukocyte recruitment, such as CCL1 and CCL2 for monocytes, CCL20 for lymphocytes, CCL21 for activated T cells, CXCL3 for neutrophils and tissue remodelling, such as ICAM1, VCAM1, MMP9 and TGFβ. Overall, the observed immune-metabolic response is characteristic of a M1 macrophage activation type [44].

**Fig. 5:**
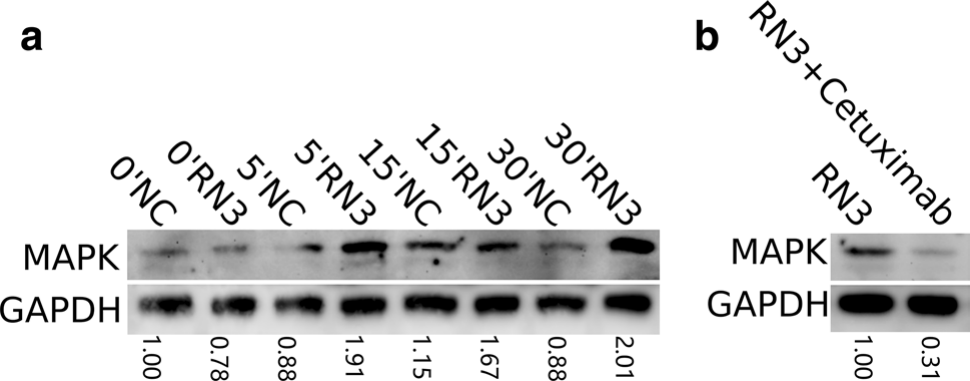
Western blot (WB) detection of pMAPK by RNase3 treatment with or without anti-EGFR Ab (Cetuximab). 1 × 10^6^ THP1 cells were plated in a 6-well culture plate and induced into macrophage by PMA treatment. **a** the phosphorylation of MAPK was detected by comparing 10 μM of RNase3 treatment for 5 min, 15 min and 30 min; **b** comparison of the phosphorylation of MAPK of the cells treated with RNase3 (RN3) for 15 min at 10 μM with and without Cetuximab pre-treatment. Ratio of pMAPK to GAPDH signal normalized to control reference value is indicated at the bottom of each lane

To explore further the involvement of the EGFR receptor in the signalling pathway activated by RNase3, we assayed the gene expression profile of selected top DEGs by qPCR at 4 and 24 h, using EGF as a positive control (Figure S2). Moreover, we evaluated the inhibitory action of a monoclonal antibody anti-EGFR (Cetuximab) on the gene expression levels. The antibody recognizes the extracellular domain of the receptor and blocks its activation. Following treatment of THP1-derived macrophages with Cetuximab, we confirmed the inhibition of the protein effect (Figure S3). Next, we confirmed the induction of the downstream pathway associated to the activation of EGFR by detection of MAP kinase (MAPK/ERK1/2) phosphorylation by western blot analysis. We observed an increase of MAPK phosphorylation as a function of time upon THP1-derived macrophages incubation with RNase 3. Besides, pre-treatment of cells with the anti-EGFR Ab, inhibited the kinase phosphorylation (Fig. 5).

### RNase3 modulates the macrophage antiviral pathway in a catalytic-dependent manner

**Fig. 6:**
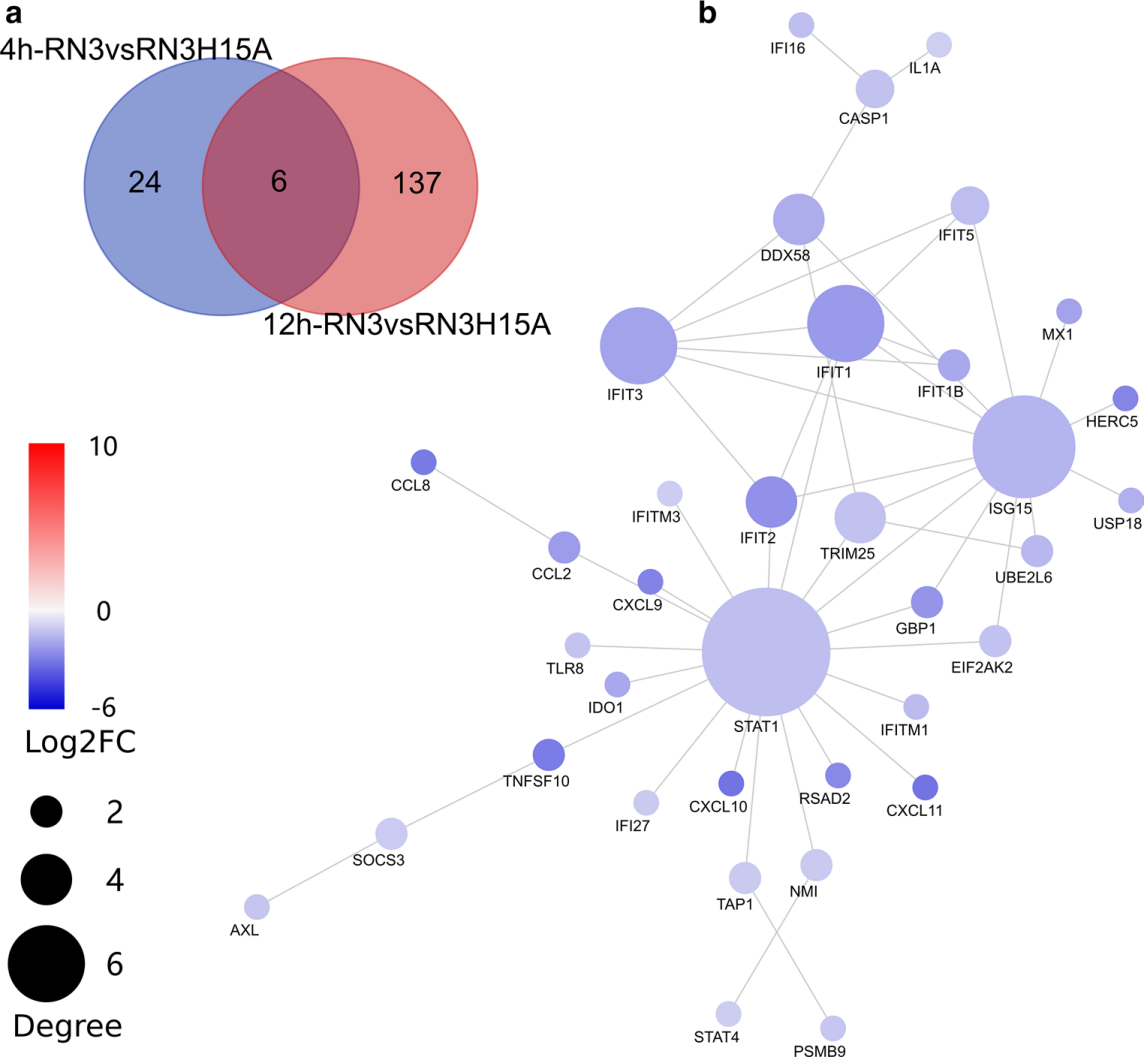
Analysis of RNase3 induced response of THP1-derived macrophages associated to catalytic activity. **a** Overlap of the DEGs identified by directly comparing the number of upregulated genes in RNase3 vs. RNase3-H15A treatment at 4 h and 12 h, respectively; **b** Protein–protein interaction (PPI) Network analysis of ribonuclease dependent protein interaction of DEGs identified by comparing RNase3 and RNase3-H15A at 12 h. The interaction between each protein pair is indicated with lines, the circle size is proportional to interaction degree. The colour bar indicated the log2FC of each gene

Although RNase3 and RNase3-H15A elicited an overall similar transcriptional response of macrophage, a direct comparison between macrophage cells treated with the wt and mutant proteins identified significant changes related to the RNase catalytic activity. Transcriptome analysis of wt versus H15A mutant revealed 30 and 143 DEGs at 4 and 12 h, respectively (Fig. 1a). Within the DEGs paired set, we found only six genes activated at both 4 and 12 h time points (Fig. 6a): *CXCL10*, *IFIT1*, *LAMTOR1*, *SOCS3*, *OASL*, and *IFIT2*. Interestingly, several of these genes are activated by the interferon pathway, such as the 2′5′ oligoadenylate synthase like protein (OASL), the CXCL10 chemokine and the interferon induced RNA binding proteins (IFIT1 and IFIT2) [45–47]. Furthermore, SOCS3 activation is usually observed following infection and plays a tissue protecting role against inflammation side effects [48].

On the other hand, when all the DEGs were analysed by connectivity and betweenness centrality, we observed 31 seed genes connected by 44 edges. The network analysis clearly pointed out to STAT1 and ISG15 as the hub genes (Fig. 6b). ISG15 expression is regulated by STAT1 transcription factor, which is characteristic of the macrophage activation by the interferon pathway and adaptation to an antiviral state. The connectivity map outlined the contribution of other interferon induced regulatory proteins, such as IFIT1-3 and the CXCL10 chemokine. By applying the KEGG pathway enrichment analysis, we mostly obtained at 12 h signalling pathways related to infection sensing and in particular associated to antiviral response (see Table 1 and Additional file 6). In contrast, no significant pathway was found when applying the DEGs at 4 h. In any case, according to GO term annotation analysis “response to virus” is the single significantly enriched at 4 h. Moreover, analysis of the DEGs at 12 h, identified diverse biological processes but in all the cases related to the cell defence response to virus (Table 2, Additional file 7). In addition, the comparative heatmap of DEGs highlighted that significant changes of wt versus H15A mutant are mostly prominent at 12 h. Overall, the results indicate that the late immune-regulatory action (12 h) induced by RNase3 dependent on the protein catalytic activity would complement the first short-term response (4 h), mostly mediated by EGFR activation. Results suggest that both RNase3-activated pathways, catalytic-dependent and independent, might work by complementing each other.

**Table 1:** KEGG pathway enrichment analysis of DEGs comparing RNase3-H15A treatment with RNase3 at 12 h

Description	Gene ratio	Bg ratio	*P* adjust	Count
Influenza A	16/82	170/7925	1.60E-09	16
Hepatitis C	13/82	155/7925	3.40E-07	13
Viral protein interaction with cytokine and cytokine receptor	10/82	100/7925	2.88E-06	10
NOD-like receptor signalling pathway	12/82	181/7925	9.50E-06	12
Cytokine-cytokine receptor interaction	14/82	294/7925	4.10E-05	14
Chemokine signalling pathway	11/82	189/7925	6.41E-05	11
Cytosolic DNA-sensing pathway	7/82	63/7925	6.41E-05	7
Toll-like receptor signalling pathway	8/82	104/7925	0.000177	8
Measles	8/82	138/7925	0.001219	8
RIG-I-like receptor signalling pathway	5/82	70/7925	0.009897	5

Significant enriched pathway list was filtered using *P* adjust < 0.01

**Table 2:** Top ten significant GO terms enriched comparing RNase3-H15A treatment with RNase3 at 12 h

Description	Gene ratio	Bg ratio	*P* adjust	Count
Defence response to virus	39/130	224/17,653	7.32E-40	39
Response to virus	42/130	310/17,653	8.42E-39	42
Negative regulation of viral process	21/130	95/17,653	9.30E-23	21
Negative regulation of viral life cycle	19/130	79/17,653	2.78E-21	19
Negative regulation of viral genome replication	16/130	55/17,653	3.21E-19	16
Regulation of multi-organism process	28/130	391/17,653	2.00E-17	28
Negative regulation of multi-organism process	21/130	172/17,653	2.00E-17	21
Regulation of viral life cycle	19/130	137/17,653	9.75E-17	19
Response to interferon-gamma	21/130	191/17,653	1.43E-16	21
Regulation of viral process	21/130	196/17,653	2.21E-16	21

### Characterization of an RNase3 overexpression THP1 cell line

**Fig. 7:**
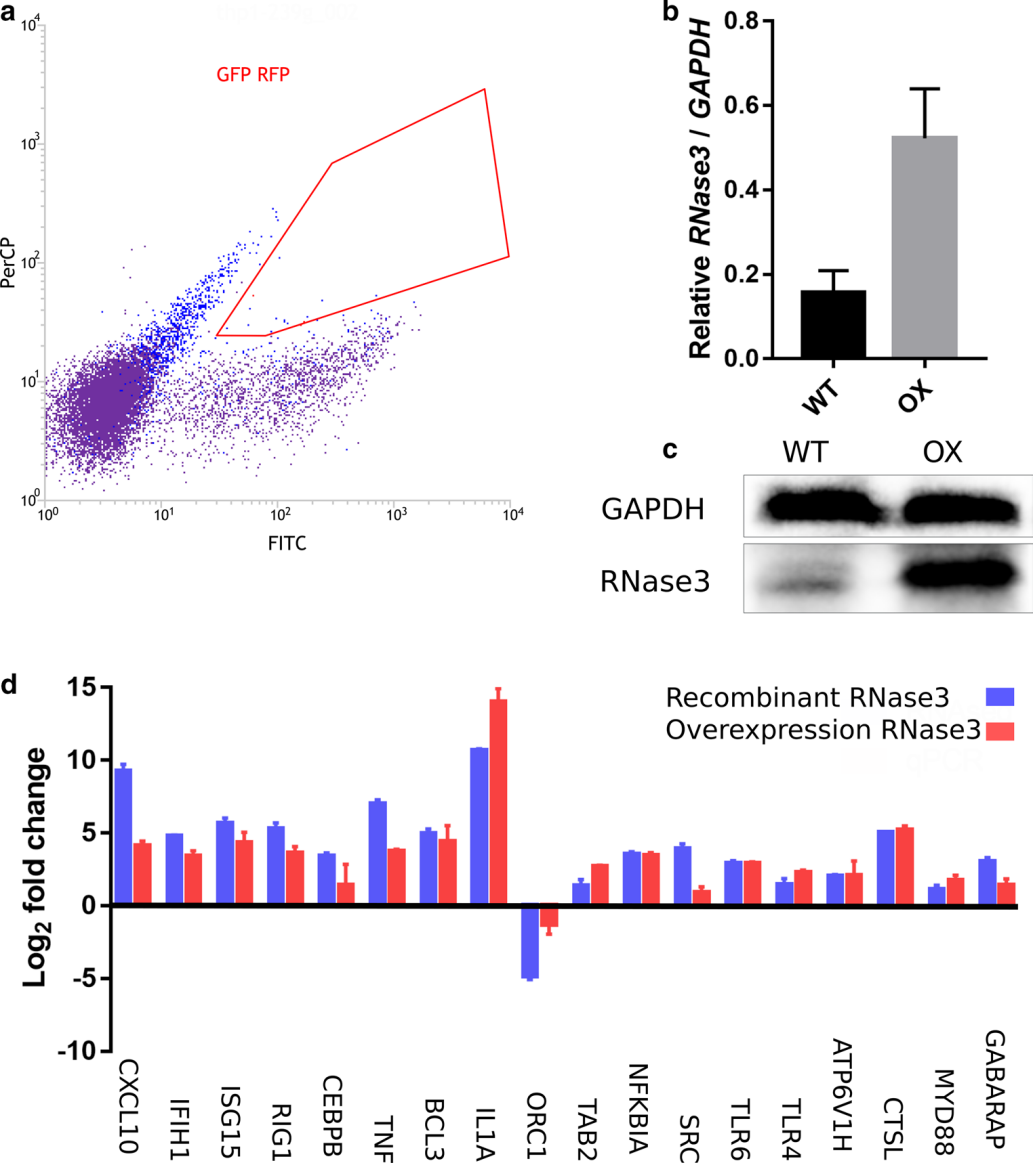
Overexpression of RNase3 in human THP1 derived macrophages. **a** FACS selection of both GFP (indicating the successful transduction of lenti239G-dCas9-VP64) and RFP (indicating the successful transduction of lenti239R-sgRNA) positive cells; **b** Comparison of the transcriptional expression of *RNase3* gene in wild-type THP1 macrophages (WT) and RNase3 overexpression THP1 macrophages (OX) by qPCR; **c** Comparison of the expression of RNase3 protein in WT and OX THP1 macrophages by WB. **d** Comparison of 18 gene expression levels between transcriptome analysis by RNAseq of THP1 macrophages treated with recombinant RNase3 and RNase3 OX THP1 macrophage cells analysed by qPCR. 18 DEGs identified from transcriptome sequencing were validated by qPCR, using WT and OX THP1-induced macrophages

To corroborate our transcriptomic results and evaluate the potential contribution in situ of the macrophage endogenous RNase3, we decided to obtain a THP1 cell line that overexpresses the protein. As quantified in our previous work, basal levels of RNase3 in the monocytic THP1 cell line are detectable but considerably low (estimated about 1/500 respect to the *GAPDH* control gene) [14]. Therefore, the overexpression of RNase3 in THP1 cells could serve to analyse the protein action within the macrophage-derived cells. CRIPSRa methodology was applied to overexpress RNase3 in THP1 cells. The plasmids encoding CRISPRa components were delivered to THP1 cells by lentiviral infection. An empty lentiviral plasmid was used for the control reference cells. The GFP and Cherry red both positive cells successfully transduced by lenti239G (encoding dCas9-VP64-GFP) and lenti239R (encoding sgRNA-Cherry red) were sorted by FACS (Fig. 7a). After induction of THP1 to macrophage, the expression of RNase3 was quantified by qPCR, observing an increase from about 1.5 to threefold in all the analysed cell lines (Figure S4). The best performance cell line (OX5) was selected for final characterization. RNase3 levels in relation to wild-type THP1 cells were quantified by both qPCR and WB, confirming a significant increase of RNase3 both at the gene and protein level (Fig. 7b, c). Besides, we confirmed that overexpression of RNase3 did not alter the macrophage cell viability, as evaluated by the MTT assay. However, the overexpression of RNase3 is significantly slowing down the duplication rate. We estimate a reduction of about 20% between 1st and 2nd day of incubation (results not shown), that might be attributed to the protein downregulation of genes associated to cell cycle (see Figs. 2 and 3).

To validate the transcriptome results obtained by NGS sequencing, we analysed the expression profile of the RNase3 overexpression THP1 cell line. A total of 18 genes were selected among the top DEGs from libraries of wild-type versus control and catalytically defective mutant versus wild-type for further qPCR analysis. The results confirmed similar expression levels induced by both endogenous and recombinant proteins (Fig. 7d).

Moreover, we compared the gene expression profile of the RNase3 overexpression THP1 cell line (OX) in the absence and presence of the anti-EGFR antibody Cetuximab and confirmed the Ab inhibition of genes associated to the EGFR pathway (Figure S5).

### Analysis of the putative RNase3-EGFR interaction by molecular modelling

**Fig. 8:**
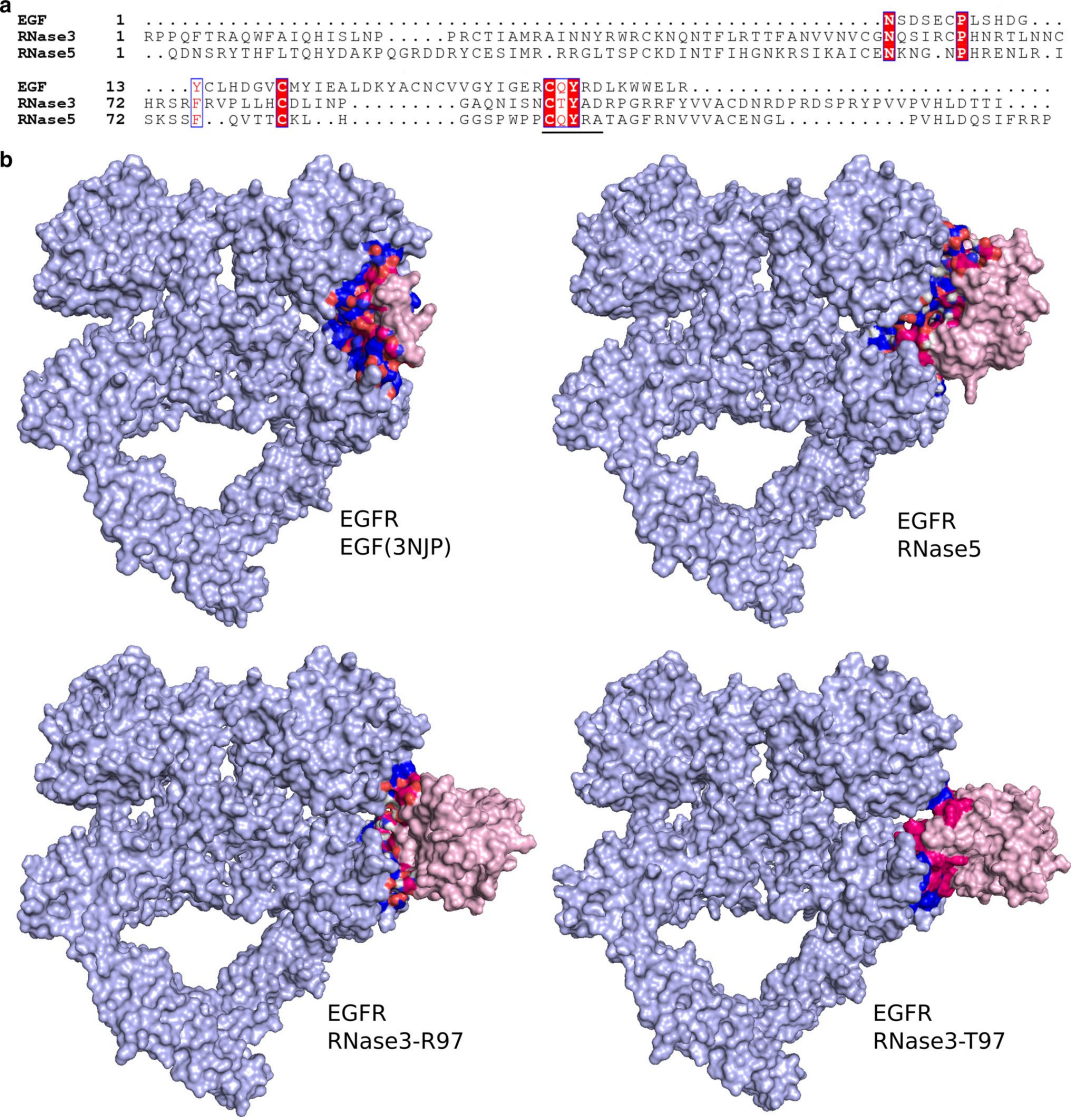
Comparison of the interaction of EGFR with RNase3 (R97 and T97), RNase5 and EGF by molecular modelling. **a** Primary sequence alignment of EGF, RNase3 and RNase5. The common interacting region is underlined. **b** Representative binding of EGFR with EGF, RNase5, and RNase3 single nucleotide polymorphisms: RNase3-R97 and RNase3-T97. The crystal complex of EGFR with EGF (PDB ID: 3NJP) is depicted. EGFR is coloured in grey, the protein ligands were coloured in pink, the interacting residues from EGFR and the ligands were coloured in blue and red, respectively

Considering that our whole transcriptome analysis pointed out to a direct activation of EGFR by RNase3, we decided to explore further the protein-receptor-binding process. Interestingly, it was recently demonstrated that RNase5/Ang, another member from the RNase A superfamily, is a direct ligand of EGFR [18]. The authors identified a C-terminal region in RNase5 needed for EGFR interaction, with significant homology to EGF binding region to the receptor. Notably, we observed how the key residues shared by RNase5 and EGF (C81, C92, Q93, Y94) are mostly conserved in RNase3 (Fig. 8a and Additional file 8). In particular, the QYRD sequence was spotted to participate in EGF interaction with the receptor by site-directed mutagenesis [49] and molecular dynamics [50]. Considering that the region of RNase5 involved in the interaction with EGFR is mostly conserved in RNase3, we decided to explore the potential protein interaction to the receptor. We applied HADDOCK software to predict the complex structures of RNase3 with the EGFR extracellular domain (Fig. 8b). The crystal structure of the EGFR-EGF complex was used as a model PDB (ID: 3NJP). A high binding affinity of RNase3 to EGFR was estimated (see Table 3). Notably, we obtained for RNase3 similar or even slightly higher binding energies, as calculated for EGF. A close inspection of the predicted complex revealed that the interaction of RNase3 with EGFR might be mediated by residues regions 1–4, 19–28, 33–35, 86–87 and 94–100. In particular, we can identify the key role of N95, R97, Y98 and D100 of RNase3, which can interact within the 417–470 region of the EGF receptor (see Additional file 8 for a full list of predicted protein–protein interactions). The molecular modelling also indicates that the RNase active site residues (H15, K38 and H128) would not be involved in the receptor binding, in agreement with our transcriptomic results. Besides, the main region identified in RNase3 overlaps with the RNase5 and EGF counterparts (Fig. 8a). However, we also observed significant differences in the main interacting region from EGF and RNases, where residues 45–51 in EGF are not conserved in RNases sequence. This suggests that the RNases mechanism is not fully equivalent to EGF functioning and might indicate a distinct target within the EGFR family. Notwithstanding, we do observed conservation of main key interacting residues, specifically within the CQYRD segment (residues 96–100 and 92–96 in RNase3 and EGF, respectively). In particular, Hung and co-workers confirmed that residues Q93 and Y94 were essential for the receptor binding to RNase5. Here, in our model for RNase3, we observed the putative binding of R97 to the receptor extracellular domain. Interestingly, RNase3 presents a single nucleotide polymorphism (SNP) at position 97 with two alternate substitutions (R/T) [51] and molecular modelling predicted slight differences in the affinity to the receptor of R97 and T97 variants (Table 3).

**Table 3:** Predicted free energies for EGFR-protein modelled complexes

	ΔG (kcal/mol)	Kd (M) at 25 °C
EGF (docking)	− 10.3	2.9·10^–8^
EGF (crystal)	− 15.8	2.5·10^–12^
RNase5	− 12.4	8.2·10^–10^
RNase3 (R97)	− 12.0	1.5·10^–9^
RNase3 (T97)	− 10.2	3.4·10^–8^

The binding affinities ΔG (kcal/mol) and dissociation constants Kd (M) were predicted using HADDOCK2.2

### Overexpression of endogenous RNase3 inhibits the macrophage intracellular infection by both M. aurum and RSV

Next, we evaluated the efficacy of the macrophage endogenous RNase3 against infection. We selected two models of macrophage intracellular infection (*M. aurum* and RSV). Both intracellular pathogens have been previously proven to be effectively eradicated within the macrophages by recombinant RNase3 addition [9, 14].

**Fig. 9:**
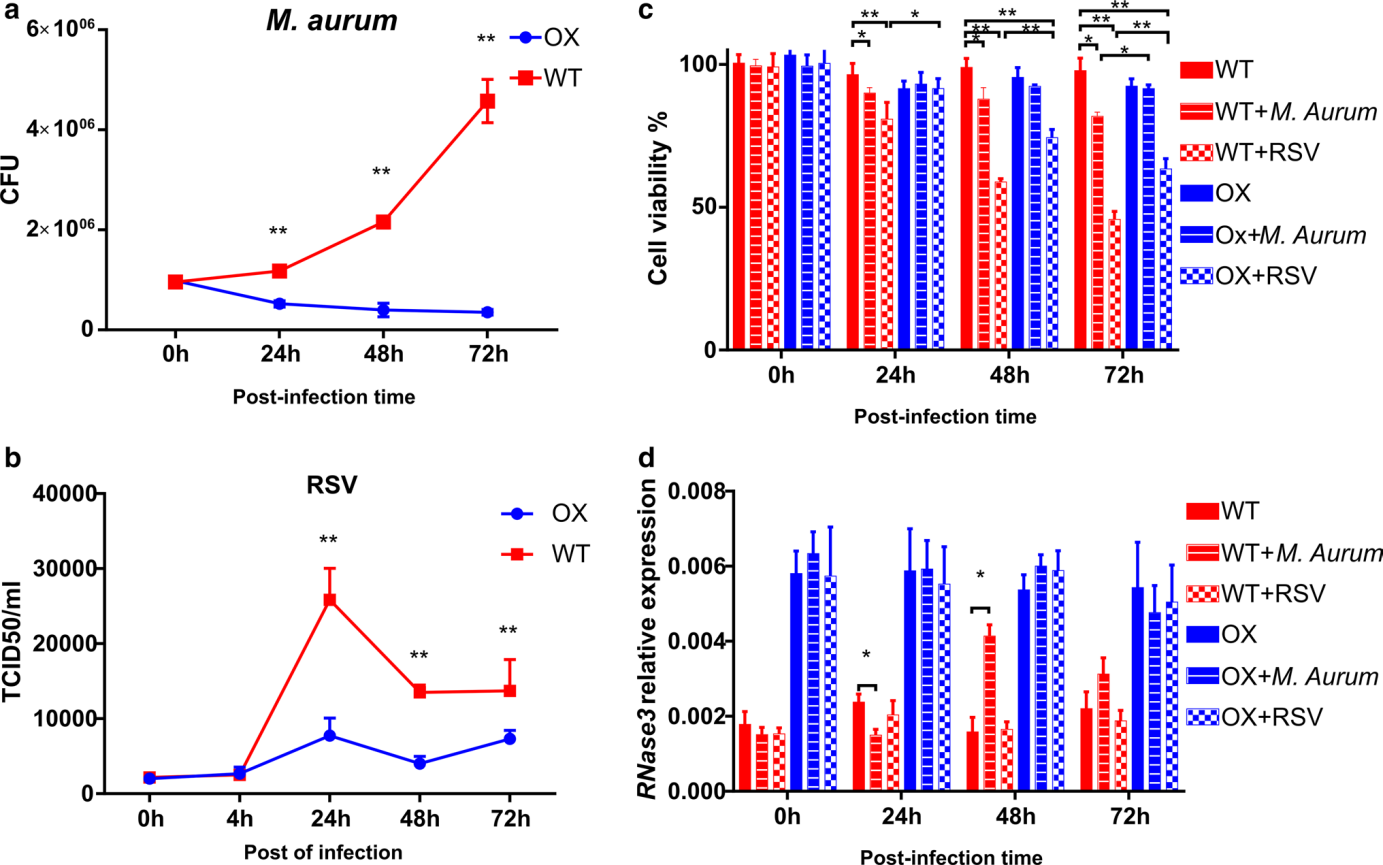
RNase3 inhibits bacterial and viral infection within macrophages. Overexpression of RNase3 in macrophage inhibits *M. aurum* and RSV proliferation. **a** the number of *M. aurum* inside of macrophage was counted by CFU assay and compared with wild-type THP1-induced macrophages (WT) and RNase3 overexpression THP1-induced macrophages (OX) for up to 3 days; **b** RSV was quantified by probe-qPCR, the intracellular RSV was normalized using *GAPDH* gene in WT and OX cells; **c** MTT assay was applied to measure the cell viability, 0 h WT group was used for normalization (100%); **d** Comparison of the relative transcriptional expression of RNase3 gene by qPCR using GAPDH as a reference. Cell viability and RNase3 expression was measured in WT, OX, WT infected with *M. aurum* (WT + *M. aurum*) or RSV (WT + RSV), and OX cells infected with *M. aurum* (OX + *M. aurum*) or RSV (OX + RSV) THP1-macrophage-derived cells; significance is indicated as *P < 0.05 and **P < 0.01

First, we infected both wild-type (WT) and RNase3-overexpression (OX) THP1 cells derived to macrophages with *M. aurum* and monitored the cell lines for 3 days. Intracellular infection was quantified by Colony Forming Unit counting (CFUs) in lysed cells after removal of extracellular mycobacteria. No significant differences in *M. aurum* initial CFUs between WT and OX cell lines were observed at the initial time point (Fig. 9a). However, *M. aurum* can easily proliferate in wild-type macrophage as indicated by an exponential CFU count increase as a function of time. On the contrary, mycobacteria cannot proliferate in the RNase3-overexpression cell line. A significant growth inhibition of *M. aurum* in RNase3 overexpression macrophages was detected at 24 h post-infection compared to WT macrophage. Noteworthy, the growth inhibition was even more prominent as the infection progressed (48 h and 72 h). The present data corroborate our previous results showing that recombinant RNase3 can efficiently mediate the eradication of macrophage intracellular infection by mycobacteria [14].

Likewise, both WT and OX THP1 cells derived to macrophages were infected with the RSV virus and the cell lines were monitored for 3 days. Probe-qPCR was applied to quantify the RSVs both extracellularly and intracellularly using an internal RSV quantification standard curve built by serially dilutions from a RSV stock (Figure S6). Starting from an equivalent RSV initial titer in both WT and OX cell lines, we observed the increase as a function of time of RSV at intracellular and extracellular levels (Fig. 9b and Fig. S7). Intracellular and extracellular RSV reached a maximum peak at 24 h and 48 h post of infection, respectively. Importantly, we found that overexpression of RNase3 in macrophage can significantly inhibit the RSV duplication at 24–72 h poi (Fig. 9b and Fig. S7). In addition, overexpression of RNase3 reduced the macrophage cell death rate caused by *M. aurum* and RSV infection (Fig. 9c).

Moreover, the transcriptional expression levels of *RNase3* were quantified in both cell lines (WT and OX) in the absence and presence of *M. aurum* and RSV infection. Overall, we confirmed that the OX cell line expresses significantly higher level of *RNase3* than WT cells at all the time points (Fig. 9d). In addition, we explored whether the cell infection by *M. aurum* and RSV could regulate the macrophage endogenous RNase3 expression. The results indicate that short-term *M. aurum* infection is downregulating RNase3 whether a long-term exposure (48–72 h) induced the protein expression. The data corroborate down- and up-regulation profile previously reported upon mycobacteria infection [14]. On the contrary, the expression of RNase3 in WT macrophage was not significantly altered by RSV infection. Furthermore, the OX cell line kept the expression levels of RNase3 stable during all the 3 days’ experiment in both *M. aurum* and RSV infection studies.

### Blockage of the EGFR receptor by Erlotinib inhibits the RNase3 antibacterial activity.

**Fig. 10:**
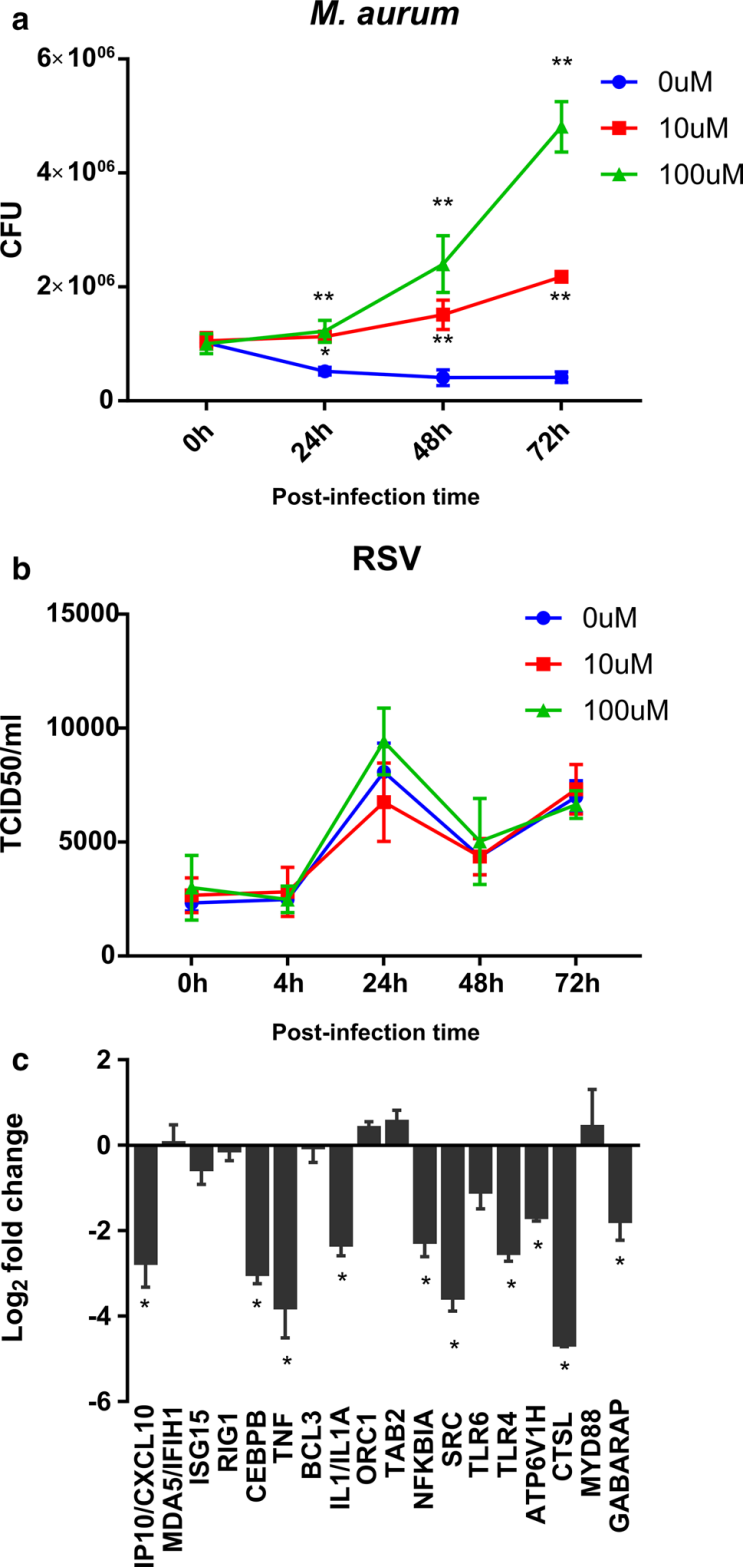
EGFR inhibitor, Erlotinib, blocks the induction of RNase3 antimycobacterial activity. Before infection, the EGFR inhibitor, Erlotinib, was used to treat RNase3 overexpression macrophages for 24 h at 0 μM, 10 μM and 100 μM. At the indicated post-infection time, the intracellular *M. aurum* (**a**) and RSV (**b**) was quantified by CFU assay and probe-qPCR assay, respectively; the median tissue culture infectious dose (TCID50) was calculated and the intracellular RSV was normalized using *GAPDH* gene. **c** The relative expression of genes was quantified by qPCR with or without Erlotinib treatment (10 μM, 24 h), and the fold change was calculated by comparing Erlotinib treatment with control. Significance is indicated as *P < 0.05 and **P < 0.01

**Fig. 11:**
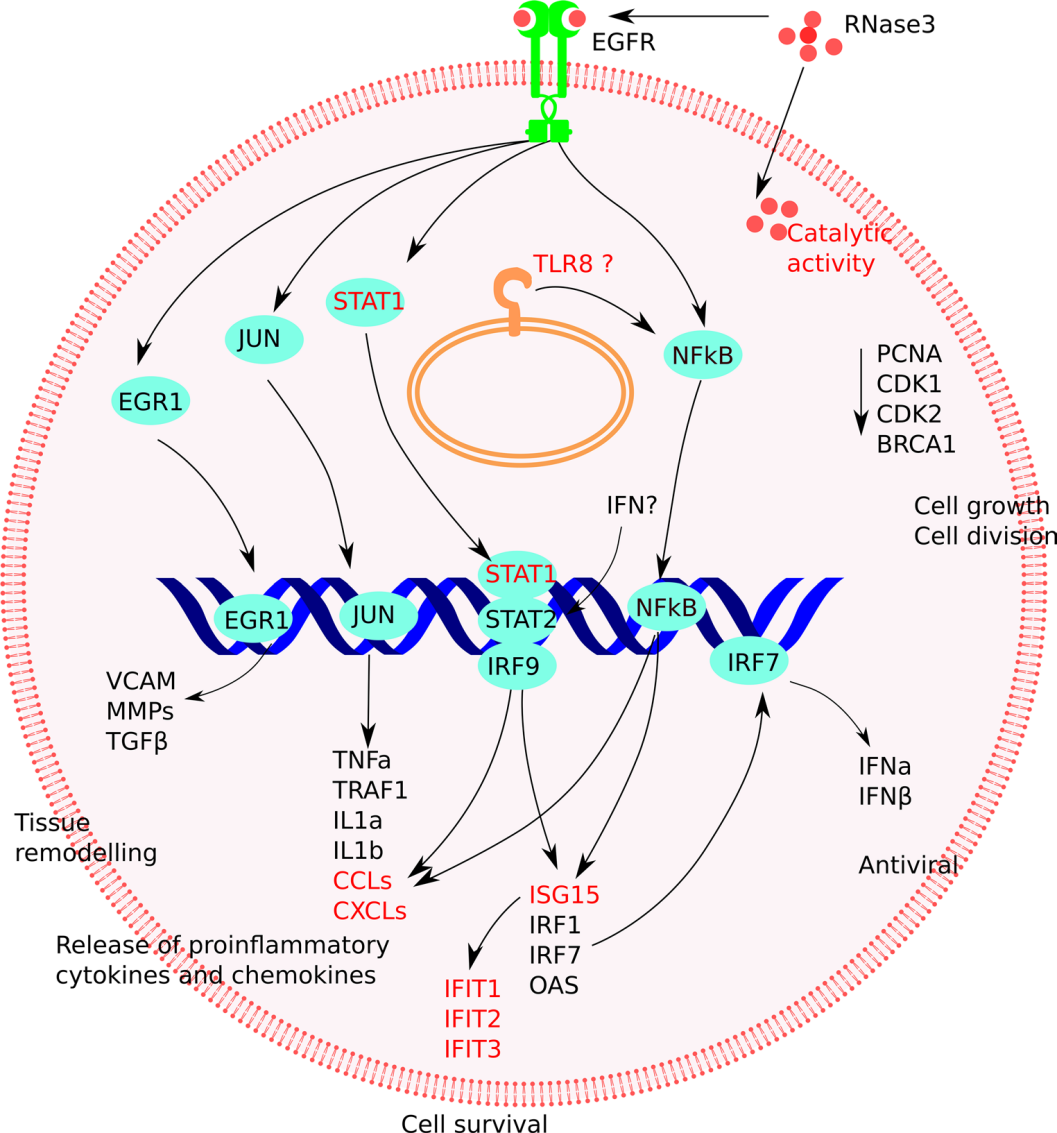
Schematic illustration of the proposed molecular mechanism of RNase3 modulation in human macrophage. The genes associated to RNase catalytic activity were labelled in red. *EGFR* epidermal growth factor receptor, *IFIT* interferon induced protein with tetratricopeptide repeats, *EGR1* early growth response 1, *JUN* jun proto-oncogene or AP-1 transcription factor subunit, *NFkB* nuclear factor Kappa B, transcription regulator, *STAT* signal transducer and activator of transcription, *IRF* interferon regulatory factor, *VCAM* vascular cell adhesion molecule, *MMP* matrix metallopeptidase, *TGF* transforming growth factor, *TNF* tumour necrosis factor, *TRAF* TNF receptor-associated factor, *IL* interleukin, *CCL* C–C motif chemokine ligand, *CXCL* C-X-C motif chemokine ligand, *ISG15* interferon induced 15 kDa protein, *OAS* 2′-5′-oligoadenylate synthetase, *IFN* interferon, *PCNA* proliferating cell nuclear antigen, *CDK* cyclin-dependent kinase, *BRCA1* Breast Cancer gene 1, DNA repair associated gene

We next assessed the effect of adding an inhibitor of EGFR activity on the observed anti-infective action of RNase3. Erlotinib inhibits EGFR activation by blocking the receptor tyrosine kinase [52]. The RNase3 overexpression macrophages were treated with Erlotinib for 24 h before infection of either *M. aurum* or RSV. As illustrated in Fig. 10a, upon addition of 10 μM of Erlotinib, we observed a significant increase of *M. aurum* growth within the macrophages. On the contrary, this scenario was not observed in RSV infection even after addition of 100 μM of Erlotinib (Fig. 10b). Moreover, we quantified and compared the fold change of a set of 18 selected genes in RNase3 overexpressed cell line in the presence and absence of Erlotinib treatment. The selected genes had previously been quantified as DEGs by both recombinant RNase3 addition and endogenous protein overexpression (see Fig. 7d). Besides, by the side-by-side comparison of the overall transcriptome between wild-type and catalytic defective RNase3, we identified the DEGs dependent or independent of the protein catalytic activity (see Additional file 1). Here, following Erlotinib treatment we observed how gene expression is uniquely altered for the genes not dependent on the protein catalytic activity (Fig. 10c). That is, addition of 10 μM of Erlotinib significantly inhibited RNase3′s induction of the expression of genes such as *CXCL10*, *CEBPB*, *TNF*, *BCL3*, *IL1*, *NFKIBA*, *SRC*, *TLR4*, *ATP6V1H*, *GABARAP*. On the contrary, *MDA5/IFIH1*, *ISG15* and *RIG1* that are dependent on RNase3′s catalytic activity, did not response to Erlotinib treatment. The results corroborated the presence of two distinct type of pathways that are regulated by RNase3 and are associated to either catalytic or non-catalytic-dependent mechanisms of action (see Fig. 11 for a schematic overview).

## Discussion

Antimicrobial peptides (AMPs) are important components of natural defence against a wide range of pathogens [53]. AMPs, originally reported to work by a direct action at the microbial cell wall, were later ascribed a diversity of immune modulatory properties that can contribute to the infection eradication [54, 55]. Human antimicrobial RNases from the RNaseA superfamily can be regarded as multifaceted AMPs that combine a microbial membrane destabilization action with immune regulatory properties [4, 56, 57]. In our recent work, we have reported how RNase3 induction of macrophage autophagy mediated the eradication of *M. aurum* intracellular infection [14]. Here, we have applied the NGS RNA sequencing methodology to explore the immune-regulatory mechanism of action of RNase3 within macrophages. The comparative transcriptomic profile of macrophages treated with wild-type and catalytic-defective RNase3 has enabled us to identify the regulation pathways related and unrelated to the protein ribonucleolytic activity. Overall, we observed that treatment with both RNase3 and RNase3-H15A triggered a common immune response. The shared transcriptome profile pattern outlined an up-regulation core response characteristic of a macrophage pro-inflammation condition [44]. Furthermore, downregulation core response indicated that the protein addition promoted the cell growth arrest and duplication inhibition (Fig. 2).

Protein–protein network analysis identified EGFR as the main hub gene together with a group of five other genes (JUN, NFkB1, STAT1, EGR1, SRC), which are on their turn directly or indirectly interacting with EGFR (Fig. 3). Furthermore, inspection of central hub down-regulated genes also revealed an association to EGFR receptor activation. Potential involvement of EGFR activation in RNase3 signalling pathway was corroborated by the blockage of the expression level of selected DEGs using Cetuximab, a monoclonal anti-EGFR Ab (Figure S3). Besides, we also confirmed the activation of MAPK phosphorylation upon cell treatment with RNase3 and its inhibition in the presence of Cetuximab (Fig. 5). Interestingly, EGFR, the epidermal growth factor receptor, is not only a key membrane receptor involved in cell survival and tissue remodelling [58, 59], but it can also mediate the macrophage activation during bacterial [60] and virus infection [61]. Specifically, EGFR signalling is critical for pro-inflammatory cytokine and chemokine production [60, 62, 63]. Therefore, our present results suggest that RNase3 mostly modulates the macrophage response via direct EGFR activation. Taking into consideration the very recent report of human RNase5 direct binding to EGFR [64], we envisaged here a structural analysis of the putative interaction of RNase3 to the receptor. Human RNase5/Ang, sharing with RNase3 a common three-dimensional structure fold, was taken as a reference to model a putative RNase3-receptor complex using the EGF-receptor structure as a common reference for both homologous RNases (Fig. 8). The results predicted a strong binding affinity of RNase3 to the EGFR extracellular domain. Molecular modelling suggested no involvement of the protein catalytic site in the receptor interaction, as indicated by our transcriptome results. Likewise, EGFR activation by RNase5/Ang was not dependent on the protein catalytic activity [18]. Interestingly, we found a high sequence homology between both RNases at the conserved key target sequence previously identified at RNase5 and EGF C-terminus [18] (Fig. 8a). In particular, structural alignment of RNase5 with the EGF molecule in complex with the extracellular domain of the receptor [65] highlighted four conserved residues: two cysteines (C81 and C92), a glutamine (Q93) and a tyrosine (Y94) [64]. C81 and C92 are conserved in most of the RNaseA family members and participate in the disulphide bonding that connects the protein N and C ends and stabilizes the overall three-dimensional structure. On their turn, Q93 and Y94 counterparts in EGF were reported to bind to the receptor [66] and were confirmed by site-directed mutagenesis to participate in RNase5 -EGFR interaction. Pairwise sequence alignment indicated that Y94 is conserved in all RNase members. On the other hand, we found partially conserved substitutions for RNase5 Q93. In particular, in RNase3 the glutamine is substituted by a threonine (T97). Interestingly, RNase3 displays a polymorphism at this position (R/T97), which has been correlated to the protein properties. In particular, R97 shows an enhanced cytotoxicity on several tested eukaryote cell lines [67]. On the contrary, the presence of a T at this position creates an N-glycosylation site and native N-glycosylated forms display a reduced cytotoxicity and antimicrobial activity [17, 68, 69]. Particular attention to R/T97 SNP in RNase3 was drawn due to the specific distribution within the population [70]. Presence of an R at 97 position has been linked to an enhanced severity of malaria and schistosomiasis side-effects [71, 72], such as hepatic fibrosis or neurological disorders, that might be associated to an overabundance of the secreted RNase3 at the infectious focus [73, 74]. The protein overproduction at the infected tissue could be detrimental to the host health, as also observed in allergic asthma and other chronic inflammatory diseases. Evolutionary studies indicated that R is the amino acid present in RNase3 lineage ancestor and is shared by most of the other primate homologues [75].

The current molecular modelling results of the protein-EGFR complex suggest the involvement of residue 97 in EGFR activation. To explore the potential contribution of the R/T97 SNP in the protein action, we compared here the recombinant non-glycosylated protein (coding for the wild-type R97) with the two glycosylated variants (R/T97). Overall, we observe a similar expression profile for all recombinant proteins. However, we identified a significant increase in the expression levels of the pro-inflammatory IL1A and NFKB1A markers as a function of the protein glycosylation degree, from the non-glycosylated form expressed in *E. coli* (RN3) to the T97 variant that incorporates an additional glycosylation site. In addition, we also confirmed that pre-treatment of macrophage cells with the anti-EGFR Ab inhibits the induced gene expression profile for all the tested recombinant proteins (Figure S8). Therefore, the qPCR data also suggest that the protein region around residue 97 is involved in the activation of EGFR related pathways. However, further studies will be required for a deep understanding of the structural underling basis of the distinct biological properties of the natural protein variants. On the other hand, the analysis of the CQYRD region within the overall vertebrate family context reveals a significant variability at position 97. A glutamine is present in RNase5/Ang and ancestral family members, shared by most fish but not amphibian RNases, whereas a high variability is found in mammalian RNases, being arginine the predominant substitution [75, 76]. The importance of this residue was highlighted by Hung and co-workers, who demonstrated that human RNase1 homologue, where position 97 is occupied by an Ala, does not activate the EGFR [18]. Additional variability is observed within the last two residues of the CQYRD region. Arginine at position 99, present at EGF and RNase5 is substituted by an Ala in some RNases, like RNase2 and RNase3 [75]. Notably, A99 in human RNase3 is conserved in all primates [75]. In addition, our modelling study identified another residue key for RNase3 interaction to the receptor, D100. An aspartic residue at position 100 is also key for EGF binding but is not present in RNase5. The residue is conserved in all RNase3 primates but absent in RNase2, its closest homologue counterpart. Therefore, structural analysis revealed subtle differences that might significantly alter the receptor recognition pattern. We can speculate that the observed variability within the interacting sequence is indicating the targeting of distinct receptor subtypes within the EGFR family. In any case, our results are in agreement with the characterization of RNase5-EGFR interaction, that discarded the contribution of the protein catalytic activity in the receptor activation [64]. In addition, the present results highlight the induction by both wild-type RNase3 and RNase3-H15A of pro-inflammatory cytokines. Complementarily, we also identified the expression of tissue remodelling proteins and chemokines, such as FN1, VCAM1, MMPs and TGFβ, in a catalytic-independent manner. The present data would reinforce the RNase3 role in tissue remodelling processes reported by in vitro wound healing models [2, 16, 77–79].

Although transcriptome analysis indicates that the majority of the macrophage response is common to both RNase3/RNase-H15A treatment, we also identified a group of genes specifically associated to the protein ribonucleolytic activity (see Fig. 6, Additional file 1). Overall, the main bulk of identified genes are related to interferon induced pathways, such as CXCL10, a leukocyte chemoattractant during viral infection, IFIT1 and 2, two antiviral RNA-binding proteins [46] and OASL [80, 81]; a 2′-5′ oligoadenylate synthetase like gene which can bind double-stranded RNA and displays antiviral activity through mediating RIG-I activation [82, 83]. Accordingly, the top-rated KEGG pathways are related to host response to virus infection (Table 2). Among them, the RIG-I-like receptors (RLRs) play a major role in pathogen sensing of RNA virus infection to initiate and modulate the antiviral immunity [84]. Importantly, not only viral RNA ligands but also processed self RNA can be detected by RLRs in the cytoplasm to trigger innate immunity and inflammation and induce gene expression against infection [84, 85]. Our network analysis identified ISG15 as a hub gene dependent on RNase3 catalytic activity (Fig. 6b). ISG15 is regarded as the hallmark gene of RIG-I like receptor-signalling pathway, which is mainly activated by viral RNA and DNA. In addition, a specific processing of the host non-coding RNA and release of dsRNA can also activate the cell intrinsic immune response through RIG-I, MDA5 or OASL sensors [85]. In addition, we observe activation of STAT1, which might be mediated by the release of specific RNA products, such as dsRNA, by RNase3. Interestingly, STAT1 is activated by both the IFN and EGFR signalling pathways. Indeed, the initial increase of IFNα and IFNβ could be induced by the NF-kB pathway, which was observed to be activated by an RNase independent manner. Following, RNase3 activity might complementary activate STAT1 and induce the further release of chemokines. Based on our data, we can hypothesize that the IFN pathway might be reinforcing the short-term signalling paths induced by a direct targeting of EGFR by RNase3 (see Fig. 11 for an illustrative scheme).

Complementarily, the RNA products can work as intercellular signalling molecules to mediate a prompt host response to infection [86]. A complex interplay between the host ncRNA and the RNA virus might determine the final outcome during infection [87]. We can speculate that RNase3 antiviral immune response may be mediated by the generation of specific RNA cleavage products. Our transcriptome results also highlighted that most of the significant DEGs related to RNase3 catalytic activity were visualized at the late (12 h) exposure time rather than at the early (4 h) time point (Figs. 4 and 6). Moreover, among the activated pathways associated to the protein ribonucleolytic we found the activation of endosomal receptors, such as the TLRs 7–9, which are associated to endosomal detection of both foreign and host signalling nucleic acid molecules [86]. On its turn, TLR8 activated by self RNAs might reinforce the NFkB pathway (see Fig. 11). In addition, the TLR9 signalling pathway is reinforced by a direct interaction and phosphorylation by the EGFR [88].

We find in the literature reference to other members of the RNaseA superfamily related to interferon-mediated response and endosomal TLR activation [89–91]. RNase7 was recently reported to activate the TLR9 signalling pathway [92, 93]. Earlier, Schein and co-workers related BS-RNase activity to a direct interaction with IFN and the cleavage of dsRNA [94, 95]. Besides, IFN is reported to activate the expression of RNase L, another RNase that belongs to a protein family totally unrelated to the RNaseA superfamily. RNase L is also induced by IFN and can release small RNA products that reinforce the IFN-mediated response [96]. Both RNase L and RNase3 induce the OAS gene, which is related to dsRNA and antiviral response pathways. The present results suggest that both protein families might cooperate to fight infection by shared convergent mechanisms of RNA processing. Indeed, recent work highlighted the importance of coordinated signalling pathways that mediate the macrophage defence mechanism against infection [97, 98]. Interestingly, a very recently work describes the participation of the human RNase2, a close relative of RNase3 sharing a 70% sequence identity, in the detection of pathogen RNA mediated by TLR8. The authors suggest that RNase2 cleavage products in synergy with RNaseT2 would act as direct ligands of TLR8 [99].

To complement our comparative transcriptome analysis, we decided to analyse in situ the potential role of the RNase3 endogenously expressed by macrophages. Characterization of a THP1-macrophage-derived cell line that overexpresses RNase3 corroborated the transcriptomic results. The observed gene expression pattern induced by RNase3 is characteristic of both EGFR- and IFN-associated pathways, which can participate in the macrophage response to bacterial and viral infection, respectively [60, 98, 100]. Moreover, our results revealed that native RNase3 expressed within the macrophages can mediate the eradication of *M. aurum* or RSV infection. Our previous work using recombinant RNases highlighted the protein antimicrobial activity against both extracellular and macrophage intracellular dwelling mycobacteria [6, 14]. The present results indicate that overexpression of endogenous RNase3 within macrophages can inhibit both *M. aurum* and RSV intracellular proliferation (Fig. 9). In addition, we observed how the mycobacteria infection is modulating the expression of RNase3, as reported for other host defence peptides [101–103]. Last, with the final aim to confirm RNase3 activation of the EGFR pathway, we treated the THP1 cell lines with Erlotinib, an inhibitor of the EGFR receptor. The receptor blockage only altered the expression profile of genes unrelated to RNase3 catalytic activity (Fig. 10). More importantly, the experimental results indicated that the receptor is required for RNase3 antibacterial but not for antiviral activity. The present data are in agreement with previous reports on RNase3 catalytic activity direct contribution on the protein antiviral [9] but not antibacterial action [14, 37, 38].

RNase3 has been reported to contribute against persistent intracellular pathogens, such as the tuberculosis bacilli or the HIV virus, that frequently coexist and threaten immune-depressed patients [98, 101]. Therefore, it is crucial to understand the protein mechanism of action against macrophage intracellular infections. Unfortunately, RNase3 pro-inflammatory action following infection might also have a detrimental effect on the host tissues. Nevertheless, the protein action at the infection focus might turn out beneficial by promoting the tissue remodelling and healing [4, 16, 78]. Besides, RNase3 induction of leukocyte recruitment should reinforce the role of other blood cell type. In particular, RNase3 is abundantly secreted by eosinophils during inflammation and infection. Upon eosinophil degranulation, pro-inflammatory cytokines and chemokines are released to the infected tissue. On its turn, the eosinophil secretory proteins can be engulfed by macrophages and participate in the eradication of intracellular infection. Therefore, the protein would mediate a positive feedback and will ensure an efficient host response. Further work is in progress to fully comprehend RNase3 signalling role. A better understanding of the regulatory pathways that mediate the host response processes induced by RNase3 should facilitate the design of alternative anti-infective drugs. The present work underlines once again the therapeutic potentiality of our own defence molecules.

## Conclusions

Comparative transcriptome profile analysis of macrophages treated with wild-type RNase3 and the catalytic-defective mutant (RNase3-H15A) revealed that the protein triggers an early pro-inflammatory response in a ribonuclease-independent manner. Moreover, protein–protein network analysis of comparative gene expression profiles indicated that the overall cell response is triggered by a direct activation of the EGFR. Interestingly, addition of an anti-EGFR antibody inhibits the induced expression of EGFR-associated genes and the phosphorylation of MAPK upon RNase3 treatment. By structural analysis, we have identified the protein region potentially involved in the receptor binding. Complementarily, comparative transcriptome analysis suggested that RNase3 catalytic activity would participate in the activation of specific pathways associated to antiviral host defence. In addition, the specific blockage of EGFR by Erlotinib indicates that the receptor-associated pathways participate in the protein antibacterial but not antiviral actions. Last, we demonstrated that endogenous overexpression of RNase3 in macrophages can inhibit *M. aurum* and RSV intracellular proliferation, which advances novel strategies in the design of alternative anti-infective drugs.

## Electronic supplementary material

Below is the link to the electronic supplementary material.

Supplementary file1 Figure S1. Principal Component Analysis (PCA) plot of Control, RNase3 and RNase3-H15A treated THP1-derived macrophage cells at 4 and 12h incubation time. Figure S2. qPCR quantified genes responded to RNase3 after 4h and 24h treatment. 1x10^6^ THP1 were plated in a 6-well culture plate and induced into macrophage by PMA treatment. Next, the cells were treated with either 50 ng/mL EGF, or 10 μM RNase3 protein during the indicated time (4h and 24h) at 37 ºC and were harvested using TRIzol reagent for RNA extraction. The relative transcriptional expression level was calculated by comparison with the housekeeping gene GAPDH. Significance is indicated as * p < 0.05. Figure S3. Anti-EGFR Ab inhibits RNase3 induction of gene expression. 1x10^6^ THP1 were plated in a 6-well culture plate and induced into macrophage by PMA treatment. Next, the cells were first treated with or without 10 μg/mL anti-EGFR Ab (Cetuximab) at 4º C for 1 h. Following, cells were washed and treated with either 50 ng/mL EGF, or 10 μM RNase3 protein during 4h at 37ºC. Then, cells were harvested using TRIzol reagent for RNA extraction. The relative transcriptional expression level was calculated by comparing with the housekeeping gene GAPDH. Significance is indicated as * p < 0.05. Figure S4. Selection of THP1/RNase3 overexpression cell lines obtained by CRISPRa. RNase3 and GAPDH genes expression were quantified by qPCR. Figure S5. Effect of overexpression of RNase3 in THP1 cells on selected marker genes, in the absence and presence of an anti-EGFR Ab inhibitor. 1x10^6^ THP1 (OX) cells were plated in a 6-well culture plate and induced into macrophage by PMA treatment. Following pre-treatement with 10 μg/mL of anti-EGFR Ab (Cetuximab) for 1 hour at 4ºC, and rest at 37ºC for 15 min, total RNA was recovered for qPCR analysis. Significance is indicated as * p < 0.05. Figure S6. Standard curve of RSV quantification by probe qPCR. A RSV stock with known concentration was serially diluted and used to build the standard curve. Figure S7. RT-PCR quantification of extracellular RSV. At each time point after infection, the supernatant of wild-type and RNase3 overexpression THP1-derived macrophages (WT and OX) was collected and the RSV virus was precipitated by the PEG6000 method and used for RNA extraction. The median tissue culture infectious dose (TCID50) was quantified by RT-PCR. Significance is indicated as ** p < 0.01. Figure S8. Comparison of gene expression profiles induced by non-glycosylated recombinant RNase3 expressed in *E. coli* (RN3) and the two glycosylated variants expressed in insect cells (R97 and T97). 3x105 THP1 were plated in a 24-well culture plate and induced into macrophage by PMA treatment. Next, the cells were first treated with or without 10 ·g/mL of anti-EGFR Ab, Cetuximab, at 4 ºC for 1 h. Following, cells were washed and treated during 4 h at 37 ºC with either 10 μM of RNase3 (RN3, non-glycosylated recombinant protein expressed in E. coli), or RNase3-Arg97 (R97) and RNase3-Thr97 (T97), which correspond to the glycosylated recombinant proteins expressed in insect cells. Cells were harvested by TRIzol reagent for RNA extraction and the relative transcriptional expression level was calculated by comparing with the housekeeping gene GAPDH. Significance is indicated as * p < 0.05 (RAR 5280 kb)

Supplementary file2 Additional file 1: Protein-coding gene list of all samples whole transcriptome: control, RNase3 and RNase3-H15A at 4 and 12h. Non-coding genes and genes with less than 10 reads (sum) were filtered out. Additional file 2: Differential expressed genes for each paired wise comparison. Additional file 3: Gene list of the RNase3 and RNase3-H15A common up and down-response genes. Additional file 4: KEGG pathway enrichment of the RNase3 and RNase3-H15A common up and down-response genes. Additional file 5: Protein-protein interaction analysis of common RNase3 and RNase3-H15A response DEGs. Additional file 6: List of DEGs enriched in cytokine-cytokine receptor interaction pathways for pairwise comparison of control, RNase3 and RNase3-H15A at 4h and 12h. Additional file 7: KEGG and GO enrichment analysis of the RNase3 vs RNase3-H15A at 4 and 12h DEGs. Additional file 8: List of interacting residues identified in EGFR -EGF, -RNase3 and -RNase5 modelled complexes (RAR 6006 kb)

Supplementary file3 Table S1. Total RNA quality. The concentration of total RNA was measured by nanodrop and the integrity of the RNA was evaluated by bioanalyzer 2100 (DOCX 21 kb)

Supplementary file4 Table S2. Oligos of sgRNA, and primers for PCR (DOCX 14 kb)

Supplementary file5 Table S3. Basic information on sequencing output and processing (DOCX 14 kb)
