# Supplementary material for: Human RNase3 immune modulation by catalytic-dependent and independent modes in a macrophage-cell line infection model
Source: Cell Mol Life Sci. 2020 Nov 23;78(6):2963–85. doi: 10.1007/s00018-020-03695-5 (PMC8004517; doi:10.1007/s00018-020-03695-5)
Supplement: Supplementary file 3 — Supplementary file3 Table S1. Total RNA quality. The concentration of total RNA was measured by nanodrop and the integrity of the RNA was evaluated by bioanalyzer 2100 (DOCX 21 kb) [file 18_2020_3695_MOESM3_ESM.docx]

**Table S1. Total RNA quality.** The concentration of total RNA was measured by nanodrop and the integrity of the RNA was evaluated by bioanalyzer 2100.

| Reference | Treatment | Type of sample | Concentration(ng/μl) | RIN |
| --- | --- | --- | --- | --- |
| A1 | Control-4h | RNA | 261 | 9.3 |
| A2 | Control-4h | RNA | 171 | 10 |
| A3 | Control-4h | RNA | 151 | 10 |
| A4 | RNase3-4h | RNA | 73 | 9.9 |
| A5 | RNase3-4h | RNA | 74 | 10 |
| A6 | RNase3-4h | RNA | 114 | 10 |
| A7 | RNase3H15A-4h | RNA | 96 | 10 |
| A8 | RNase3H15A-4h | RNA | 81 | 10 |
| A9 | RNase3H15A-4h | RNA | 90 | 10 |
| A10 | Control-12h | RNA | 78 | 10 |
| A11 | Control-12h | RNA | 66 | 10 |
| A12 | Control-12h | RNA | 61 | 10 |
| A13 | RNase3-12h | RNA | 66 | 10 |
| A14 | RNase3-12h | RNA | 65 | 10 |
| A15 | RNase3-12h | RNA | 101 | 8.8 |
| A16 | RNase3H15A-12h | RNA | 77 | 10 |
| A17 | RNase3H15A-12h | RNA | 80 | 10 |
| A18 | RNase3H15A-12h | RNA | 80 | 10 |
