# Supplementary material for: Human RNase3 immune modulation by catalytic-dependent and independent modes in a macrophage-cell line infection model
Source: Cell Mol Life Sci. 2020 Nov 23;78(6):2963–85. doi: 10.1007/s00018-020-03695-5 (PMC8004517; doi:10.1007/s00018-020-03695-5)
Supplement: Supplementary file 4 — Supplementary file4 Table S2. Oligos of sgRNA, and primers for PCR (DOCX 14 kb) [file 18_2020_3695_MOESM4_ESM.docx]

**Table S2. Oligos of sgRNA, and primers for PCR**

| Category | Name | Sequence |
| --- | --- | --- |
| Guide RNA | sgRNA1 | GGCAGCTGGTCTCCCCTACTTGG |
|  | sgRNA2 | AGAGCAGCAGGGCTGTCCTTGGG |
|  | sgRNA3 | TCAATCTGTGGGTTTCTGCATGG |
|  | sgRNA4 | AAAAAGGATGATTGCACAAGTGG |
|  | sgRNA5 | TGTTTAAATAAAGCTTCCCTTGG |
| Sequencing Primer | U6-seq | GAGGGCCTATTCCCATGATT |
| PCR primers | CXCL10-Fw | GCCATTCTGATTTGCTGCCT |
|  | CXCL10-Rv | GCAGGTACAGCGTACAGTTCT |
|  | IFIH1-Fw | GCATATGCGCTTTCCCAGTG |
|  | IFIH1-Rv | CTCTCATCAGCTCTGGCTCG |
|  | ISG15-Fw | GCGCAGATCACCCAGAAGAT |
|  | ISG15-Rv | GTTCGTCGCATTTGTCCACC |
|  | RIG1-Fw | TGATTGCCACCTCAGTTGCT |
|  | RIG1-Rv | CTGCTTTGGCTTGGGATGTG |
|  | CEBPB-Fw | AAGCACAGCGACGAGTACAA |
|  | CEBPB-Rv | CCCCAAAAGGCTTTGTAACCA |
|  | TNF-Fw | CTGGGCAGGTCTACTTTGGG |
|  | TNF-Rv | CTGGAGGCCCCAGTTTGAAT |
|  | BCL3-Fw | GCCTACACCCCTATACCCCA |
|  | BCL3-Rv | GATGTCGATGACCCTGCGG |
|  | IL1A-Fw | TGAGCTCGCCAGTGAAATGA |
|  | IL1A-Rv | AACACGCAGGACAGGTACAG |
|  | ORC1-Fw | AAGCTTTGGAGCCGGCCAT |
|  | ORC1-Rv | TGATCTCCGAGAAGGCCACT |
|  | TAB2-Fw | TACGAATGGCCCAAGGAAGC |
|  | TAB2-Rv | CACAGCAGGCATCCAGGTTA |
|  | NFKBIA-Fw | TGTGCTTCGAGTGACTGACC |
|  | NFKBIA-Rv | TCACCCCACATCACTGAACG |
|  | SRC-Fw | AACAAGTGCGGCCATTTCAC |
|  | SRC-Rv | GGAGTTGAAGCCTCCGAACA |
|  | TLR6-Fw | GCAGGGGACAATCCATTCCA |
|  | TLR6-Rv | AGAATCAGGCCAGCCCTCTA |
|  | TLR4-Fw | TGCCGTTTTATCACGGAGGT |
|  | TLR4-Rv | GGGCTAAACTCTGGATGGGG |
|  | ATP6V1H-Fw | GAGACGCCTACTTGGCTCTGA |
|  | ATP6V1H-Rv | TTCAACAGCAACACCGCTAC |
|  | CTSL-Fw | CTGCTGGCCTTGAGGTTTTA |
|  | CTSL-Rv | GCAGCCTTCATTGCCTTGAG |
|  | MYD88-Fw | ACCCAGCATTGGTGCCG |
|  | MYD88-Rv | GGTTGGTGTAGTCGCAGACA |
|  | GABARAP-Fw | CTCCCTTATTCAGGACCGGC |
|  | GABARAP-Rv | TGCCAACTCCACCATTACCC |
| RSV-A | RSV-A-Probe | TCCCATTATGCCTAGGCCAGCAGCA |
|  | RSV-A-Fw | CTCAATTTCCTCACTTCTCCAGTGT |
|  | RSV-A-Rv | CTTGATTCCTCGGTGTACCTCTGT |
