# Supplementary material for: Human RNase3 immune modulation by catalytic-dependent and independent modes in a macrophage-cell line infection model
Source: Cell Mol Life Sci. 2020 Nov 23;78(6):2963–85. doi: 10.1007/s00018-020-03695-5 (PMC8004517; doi:10.1007/s00018-020-03695-5)
Supplement: Supplementary file 5 — Supplementary file5 Table S3. Basic information on sequencing output and processing (DOCX 14 kb) [file 18_2020_3695_MOESM5_ESM.docx]

**Table S3. Basic information on sequencing output and processing**

| Reference | Treatment | Total reads | Total raw base pair(fastq) | Sam base | Mapping reads percentage |
| --- | --- | --- | --- | --- | --- |
| A1 | Control-4h | 28433026 | 4.2GB | 7.8GB | 96.69% |
| A2 | Control-4h | 28164308 | 4.3GB | 7.7GB | 96.97% |
| A3 | Control-4h | 28470445 | 4.3GB | 7.8GB | 97.09% |
| A4 | RNase3-4h | 28535014 | 4.3GB | 7.5GB | 96.69% |
| A5 | RNase3-4h | 27749512 | 4.2GB | 7.3GB | 96.36% |
| A6 | RNase3-4h | 28306352 | 4.3GB | 7.5GB | 96.63% |
| A7 | RNase3H15A-4h | 28752432 | 4.3GB | 7.6GB | 96.81% |
| A8 | RNase3H15A-4h | 28535256 | 4.3GB | 7.5GB | 96.77% |
| A9 | RNase3H15A-4h | 28373650 | 4.3GB | 7.6GB | 96.53% |
| A10 | Control-12h | 28163110 | 4.3GB | 7.7GB | 96.92% |
| A11 | Control-12h | 28508004 | 4.3GB | 7.8GB | 96.65% |
| A12 | Control-12h | 28581025 | 4.4GB | 7.8GB | 96.84% |
| A13 | RNase3-12h | 27864433 | 4.3GB | 7.3GB | 96.59% |
| A14 | RNase3-12h | 28659039 | 4.3GB | 7.5GB | 95.16% |
| A15 | RNase3-12h | 28791962 | 4.4GB | 7.8GB | 96.79% |
| A16 | RNase3H15A-12h | 28618105 | 4.3GB | 7.6GB | 96.65% |
| A17 | RNase3H15A-12h | 28341850 | 4.3GB | 7.5GB | 96.81% |
| A18 | RNase3H15A-12h | 28456211 | 4.3GB | 7.6GB | 96.51% |
